# Harnessing fungal nonribosomal cyclodepsipeptide synthetases for mechanistic insights and tailored engineering†
†Electronic supplementary information (ESI) available: Detailed methodology, alignments, cloning, purification, NMR, bioactivity. See DOI: 10.1039/c7sc03093b



**DOI:** 10.1039/c7sc03093b

**Published:** 2017-09-25

**Authors:** Charlotte Steiniger, Sylvester Hoffmann, Andi Mainz, Marcel Kaiser, Kerstin Voigt, Vera Meyer, Roderich D. Süssmuth

**Affiliations:** a Fachgebiet Biologische Chemie , Institut für Chemie , Technische Universität Berlin , Strasse des 17. Juni 124 , 10623 Berlin , Germany . Email: roderich.suessmuth@tu-berlin.de; b Parasite Chemotherapy , Medical Parasitology & Infection Biology , Swiss Tropical and Public Health Institute , Socinstrasse 57 , 4051 Basel , Switzerland; c University of Basel , Petersplatz 1 , 4003 Basel , Switzerland; d Jena Microbial Resource Collection (JMRC) , Leibniz-Institut für Naturstoff-Forschung und Infektionsbiologie , Hans-Knöll-Institut , Adolf-Reichwein-Straße 23 , 07745 Jena , Germany; e Fachgebiet Angewandte und Molekulare Mikrobiologie , Institut für Biotechnologie , Technische Universität Berlin , Gustav-Meyer-Allee 25 , 13355 Berlin , Germany

## Abstract

Nonribosomal peptide synthetases represent potential platforms for the design and engineering of structurally complex peptides. While previous focus has been centred mainly on bacterial systems, fungal synthetases assembling drugs like the antifungal echinocandins, the antibacterial cephalosporins or the anthelmintic cyclodepsipeptide (CDP) PF1022 await in-depth exploitation. As various mechanistic features of fungal CDP biosynthesis are only partly understood, effective engineering of NRPSs has been severely hampered. By combining protein truncation, *in trans* expression and combinatorial swapping, we assigned important functional segments of fungal CDP synthetases and assessed their *in vivo* biosynthetic capabilities. Hence, artificial assembly line components comprising of up to three different synthetases were generated. Using *Aspergillus niger* as a heterologous expression host, we obtained new-to-nature octa-enniatin (4 mg L^–1^) and octa-beauvericin (10.8 mg L^–1^), as well as high titers of the hybrid CDP hexa-bassianolide (1.3 g L^–1^) with an engineered ring size. The hybrid compounds showed up to 12-fold enhanced antiparasitic activity against *Leishmania donovani* and *Trypanosoma cruzi* compared to the reference drugs miltefosine and benznidazole, respectively. Our findings thus contribute to a rational engineering of iterative nonribosomal assembly lines.

## Abstract 2

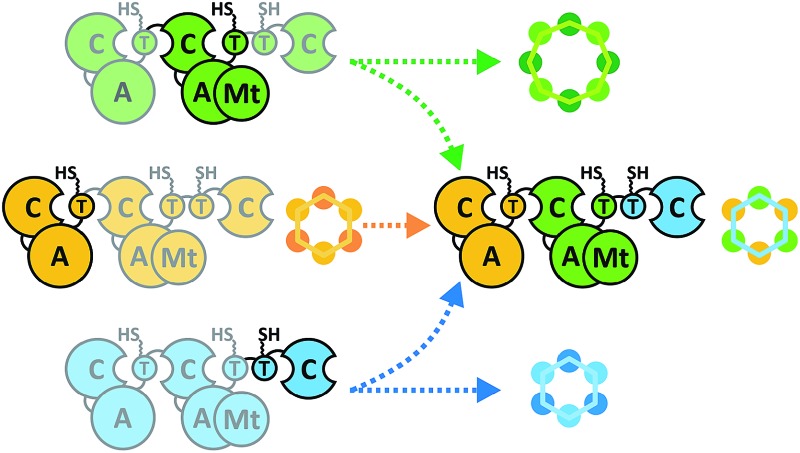
Hybrid fungal CDP synthetases are constructed from three different origins to produce highly active cyclodepsipeptides up to g L^–1^ scale.

## Introduction

**Fig. 1:**
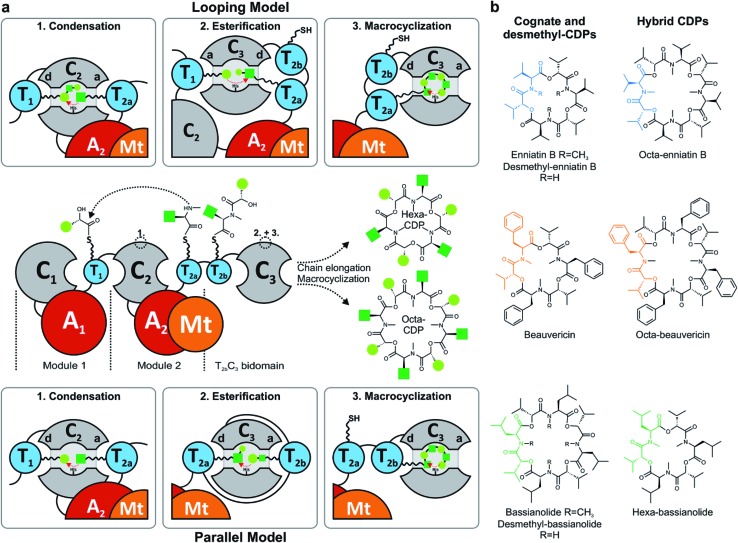
Alternative CDP biosynthesis models and structures. (a) Models of CDP biosynthesis. Fungal CDP synthetases produce six- and eight-membered CDPs, with module 1 incorporating d-HAs and module 2 activating l-AAs. The building blocks are coupled until the chain length is long enough to be cyclized by the C_3_ domain. In the looping model (top), the depsipeptide chain grows by attachment of a single building block (HA or AA), shuttling between T_1_ and T_2a/b_. The role of T_2b_ is still unclear. In the parallel model (bottom), the depsipeptide chain grows by addition of dipeptidols. a: acceptor site, d: donor site. (b) Cognate, non-methylated and hybrid CDP products. Iterative units are colored.

Nonribosomal peptide synthetases (NRPSs) assemble a great variety of linear and cyclic peptides, which display a diverse spectrum of valuable pharmacologically useful bioactivities. Amongst those biosynthetic machineries, iterative-type NRPSs from filamentous fungi are a remarkable subgroup, which produce *N*-methylated cyclodepsipeptides (CDPs) of different ring sizes. Hexa-CDPs such as enniatin B (*Fusarium* ssp.) and beauvericin (*Beauveria bassiana*) as well as octa-CDPs like PF1022A (*Rosellinia* sp.) and bassianolide (*B. bassiana*) (Fig. 1b) show various antibacterial, anticancer, anthelmintic and insecticidal activities.^1^ This turns them into promising study candidates for further drug discovery and development. Iterative CDP synthetases consist of two modules, each containing domains for adenylation (A), transfer (T) and condensation (C) of the substrate, as well as a terminal T_2b_–C_3_ bidomain (Fig. 1a). The first module (M_1_) activates d-α-hydroxycarboxylic acids (d-HA) and has been shown to be promiscuous concerning aliphatic and aromatic d-HAs to varying extents.^2–4^ The d-HA is subsequently coupled to an l-α-amino acid (l-AA), which is activated and *N*-methylated by the second module (M_2_) containing an auxiliary *N*-methyltransferase (Mt) domain. Module 2 has also been reported to process different *N*-Me-l-amino acid (*N*-Me-l-AA) building blocks to a certain degree.^5,6^ Concerning substrate elongation, it has been proposed that the growing depsipeptide chain is elongated either in dipeptidol units (parallel mode)^7,8^ or in a stepwise fashion using single HA or AA building blocks (linear mode; Fig. 1a).^9^ Recently, the linear mode has been strongly favored, stating that the growing chain is shuttling between the T_1_ and T_2a/b_ domains which alternately switch their donor and acceptor roles.^10^ For efficient macrocyclization of the full-length linear depsipeptide precursor, the N-terminal C_1_ and C-terminal C_3_ domain have previously been proposed to interact by forming a cavern.^9^ In contrast, the C_3_ domain alone was very recently found to mediate macrocyclization,^10^ which is in agreement with the established function of so-called termination C (C_term_) domains in fungal linear NRPSs.^11^ Still, various biosynthetic features of CDP synthetases are only partially understood, including mechanistic insights into how hexa- and octa-CDPs are assembled.

Due to their modular architecture and high homology, CDP synthetases appear well suited to study domain-specific contributions by combinatorial exchanges, while generating novel peptide structures. Consequently, fungal NRPSs offer an excellent opportunity for engineering approaches. Inspiration comes from various engineering attempts in bacterial systems, including module fusion,^12^ substitution,^13^ extension^14^ and deletion^15^ of NRPS segments. Initial attempts in fungal NRPS-engineering have shown encouraging results *in vivo*, leading to novel nonribosomal peptides (NRPs) including derivatives of enniatin and beauvericin with altered d-HA side chains.^16–18^ Since the chemical synthesis of CDPs has some drawbacks and limitations, biotechnological production in heterologous microbial cell factories is a promising alternative for sustainable CDP production.^17,19^ However, as long as our mechanistic understanding of fungal NRPS assembly remains limited, an effective engineering of fungal iterative NRPSs is unfeasible. We thus focused on the functional analysis of various catalytic steps of CDP synthetases, whilst concomitantly establishing an *in vivo* expression system for new-to-nature CDP derivatives with altered bioactivities. While we were preparing this manuscript, Yu *et al.* proposed a linear CDP biosynthesis model, which complements our findings.^10^

## Experimental

### Selection and design of swapping and truncation sites

Structural information about flexible T–C linker regions was obtained from the T_5_C_6_ bidomain of tyrocidine synthetase from *Brevibacillus brevis* (TycC_5–6_; PDB ; 2JGP).^20^ Using the TycC_5–6_ T–C bidomain as a template, structural models of EnSYN-, BeSYN- and BaSYN-T_1_–C_2_ and T_2b_–C_3_ bidomains were generated (SWISS-MODEL^21^). The homology models were aligned to TycC_5–6_ (PyMOL, Schrödinger, LLC) to identify promising swapping sites in the linker regions. The structurally identified swapping regions were annotated in a multiple sequence alignment with the synthetases PF-, En-, Be- and BaSYN in order to define the distinct T_1_–C_2_ and T_2b_–C_3_ swapping sites (ESI Fig. 1†). The latter site was also used for the construction of EnSYNΔC_3_ (ESI Fig. 2†). For T_2a_–T_2b_ swapping, the determined T_2b_–C_3_ swapping site was annotated for the T_2a_–T_2b_ linker region in a multiple sequence alignment. Structural information about the C_3_ domain was obtained from the T–C_term_ bidomain of TqaA from *Penicillium aethiopicum* (PDB ; 5EJD, ESI Fig. 3†).^22^ Based on TqaA–C_term_, the borders of the two subdomains C_NTD_ and C_CTD_ were defined (ESI Fig. 4†) and annotated in a multiple sequence alignment with PF-, En-, Be- and BaSYN (ESI Fig. 5†) in order to define the distinct C_3sub_-swapping sites (ESI Fig. 1†).

For construction of the SYNΔC_1_ constructs, structural information about the C–A linker region was obtained from the termination module (C–A–T–Te) of surfactin A-synthetase from *Bacillus subtilis* (SrfA-C; PDB ; 2VSQ).^23^ The identified truncation region was annotated in a multiple sequence alignment with PF-, En-, Be- and BaSYN to define the distinct C_1_ truncation site (ESI Fig. 2†). For truncation of the Mt domain insertion, the boundaries were identified in a multiple sequence alignment of the PF-, En-, Be- and BaSYN-A_1_ and -A_2_ domains as well as the gramicidin A synthetase A domain (GrsA).^24^ Structural information about A domains was obtained from the crystal structure of GrsA (PDB ; 1AMU)^24^ to identify secondary structure elements flanking the loop region in which Mt domains are commonly embedded. The loop region was annotated in a multiple sequence alignment of the PF-, En-, Be- and BaSYN-A_2_ domains to identify the distinct truncation sites (ESI Fig. 2†). Based on GrsA, structural models of the EnSYN- and BaSYN-A_1_ domain and truncated A_2_ domains were generated (SWISS-MODEL) and aligned (PyMOL) to confirm the construction of a loop in A_2_ similar to A_1_ (ESI Fig. 6†). Further experimental details are provided in the ESI.†

## Results and discussion

### Providing the basis for an efficient fungal NRPS engineering

**Fig. 2:**
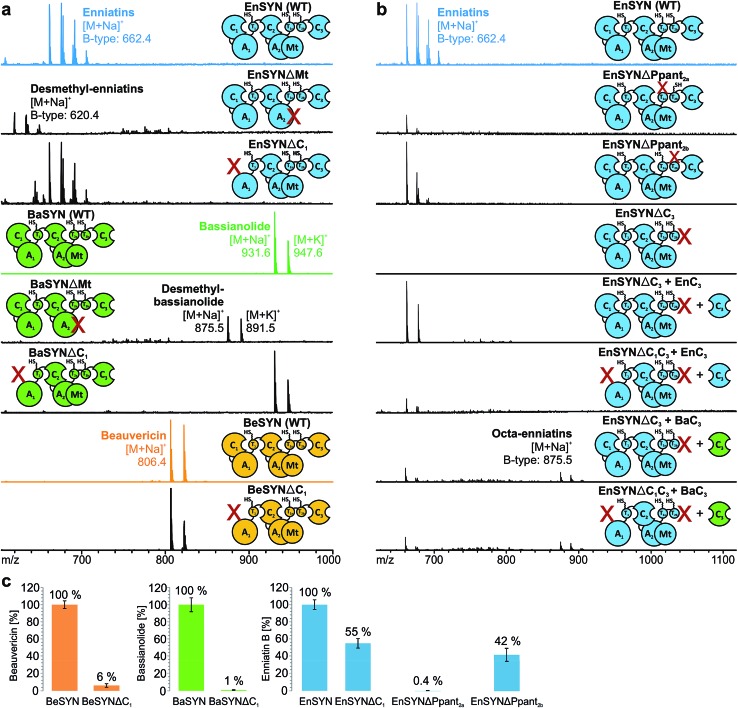
Mechanistic studies on fungal CDP synthetases. *In vivo* CDP production of (a) wild-type, mutated (EnSYNΔPpant_2a/b_) and truncated synthetases (SYNΔC_1_, SYNΔMt) as well as (b) *in trans* combinations synthesized in *E. coli* and monitored by MALDI-TOF-MS. (c) CDP yields in *E. coli* strains containing wild-type, ΔC_1_- and ΔPpant_2a/b_-synthetases determined by LC-ESI-MS (*n* = 3 cultures, standard deviation).

Due to low synthesis yields, many engineered *in vivo* NRPS systems are of limited value for deriving extensive conclusions on mechanisms or for a sustainable production.^13,14,17^ In order to lay a robust foundation for engineering approaches, we investigated the individual contributions of the C_1_, C_3_, T_2a/b_ and Mt domains to CDP biosynthesis (Fig. 1a). A comparison of the seven conserved core motifs of canonical C domains^25^ indicated that they all differ significantly between the C_1_, C_2_ and C_3_ domains, suggesting different roles in CDP biosynthesis (ESI Tables 1–3†). This inspired us to construct truncated versions of the wild-type enniatin synthetase (EnSYN), beauvericin synthetase (BeSYN) and bassianolide synthetase (BaSYN) lacking the C_1_ domain. All expression assays were performed *in vivo* in *Escherichia coli* (DE3), harboring the fungal phosphopantetheinyl transferase (PPTase) *npgA* derived from *A. nidulans* DM3365 on a second plasmid. The co-expressed NpgA displayed a 14-fold enhanced production of the fungal CDP products in comparison to the endogenous PPTase and 4.8-fold increase when compared to Sfp (ESI Fig. 7†). Crude ethyl acetate extracts of the cell pellets were analyzed by MALDI-TOF-MS and LC-ESI-MS. Unlike previous approaches in *Saccharomyces cerevisiae*,^10^ the GST-tagged C_1_ deletion constructs (SYNΔC_1_; ∼324 kDa) were successfully produced in *E. coli* co-expressing NpgA (ESI Fig. 8†). Most importantly, all SYNΔC_1_ constructs maintained production of their cognate CDPs (Fig. 2a), while production yields dropped to 1–55% compared to the wild-type enzymes (Fig. 2c). This clearly indicated that the C_1_ domain plays an important role in CDP assembly, but that, in contrast to the cavern model,^9^ the C_1_ domain is not involved in macrocyclization.

In accordance with a recent *in trans* expression of the BeSYN-C_3_ domain,^10^ we generated truncated versions of EnSYN lacking the C_3_ domain (EnSYNΔC_3_) and monitored *in vivo* production of enniatins with and without co-expression of the excised EnSYN-C_3_ domain. EnSYNΔC_3_ was unable to produce enniatins, however, CDP production could be restored *in trans* by co-expression with the free-standing EnSYN-C_3_ domain (Fig. 2b). The EnSYN-C_3_ domain could also restore enniatin production of a synthetase construct devoid of both the C_1_ and C_3_ domains (EnSYNΔC_1_C_3_; Fig. 2b). Surprisingly, a combination of EnSYNΔ(C_1_)C_3_ with a heterologous C_3_ domain derived from the octa-CDP-producing BaSYN even enabled the production of new-to-nature octa-enniatin B (comprising one d-Hiv-Val unit more; Fig. 2b). To the best of our knowledge, octa-enniatin has not been reported to date. Hence, the function of the C_3_ domain can clearly be assigned to macrocyclization and ring size determination, which supports recent findings.^10^ Additionally, we aimed at challenging the flexibility of fungal CDP synthetases concerning non-methylated AAs. Therefore, we constructed variants of EnSYN and BaSYN devoid of the Mt domain (EnSYNΔMt, BaSYNΔMt; ESI Fig. 6†). Indeed, EnSYNΔMt and BaSYNΔMt exclusively produced the non-methylated analogues desmethyl-enniatin and desmethyl-bassianolide, respectively (Fig. 2a). Notably, these compounds represent the first example of a directed *in vivo* production of CDPs lacking backbone methylation. The findings are reminiscent of natural enniatins produced by EnSYN with only one or two *N*-methylations (type B2, B3), while for BaSYN, no natural non-methylated species have been reported so far. Finally, we challenged the linear CDP elongation model similar to a recent BeSYN-based approach^10^ by constructing versions of EnSYN with mutations at the conserved serines bearing the phosphopantetheine groups Ppant_2a_ (S2538A) or Ppant_2b_ (S2632A). Like the BeSYN-variants, both mutants (EnSYNΔPpant_2a_, EnSYNΔPpant_2b_) were still able to produce their wild-type CDP enniatin *in vivo* in *E. coli* (Fig. 2b). Similar to recent findings,^10^ the production of enniatin B by EnSYNΔPpant_2a_ dropped to 0.4%, whereas EnSYNΔPpant_2b_ still produced 42% compared to the wild-type synthetase (Fig. 2c). This observation confirms that the use of T_2a_ or T_2b_ as a waiting position is very unlikely, thus strongly supporting the linear/looping model.

### Active chain-length control for hybrid CDP production

**Fig. 3:**
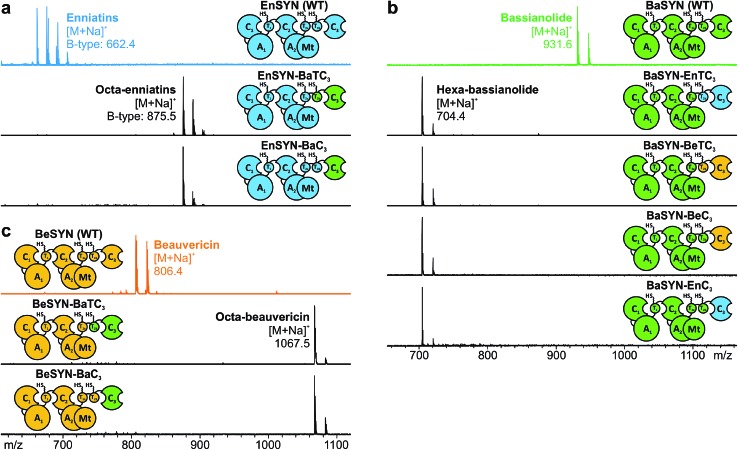
Structural diversification of fungal CDPs. *In vivo* CDP production of wild-type and hybrid synthetases with swapped C-terminal part (see color code) based on EnSYN (a), BaSYN (b) and BeSYN (c) synthesized in *E. coli* and monitored by MALDI-TOF-MS.

The identification of the C_3_ domain as the ring size determining domain in fungal iterative NRPSs^10^ inspired us to design new-to-nature CDPs of tailored ring size. To this end, we generated a set of hybrid synthetases based on the hexa-CDP-producing EnSYN and BeSYN as well as the octa-CDP producing BaSYN by exchanging the C_3_ domain or the T_2b_–C_3_ bidomain. This rendered XSYN-YC_3_ and XSYN-YTC_3_ constructs with X and Y referring to the NRPS core and the complementary heterologous segment, respectively (Fig. 3). Based on the identified domain boundaries in multiple sequence and structure alignments (see Experimental section), the domain swapping was performed in the inter-domain linker regions (ESI Fig. 1†). The resulting GST-tagged hybrid synthetases (∼375 kDa) were produced in soluble form in *E. coli* co-expressing NpgA (ESI Fig. 8†). Indeed, the hybrid synthetases EnSYN–BaTC_3_ and EnSYN–BaC_3_ both efficiently produced octa-enniatin B (4× *N*-Me-l-Val, *m*/*z* 853.553 [M + H]^+^; Fig. 3a). In analogy, the hybrids BeSYN–BaTC_3_ and BeSYN–BaC_3_ produced octa-beauvericin (*m*/*z* 1045.553 [M + H]^+^; Fig. 3c). So far, octa-beauvericin was only known from total synthesis^26^ and the *in vivo* biosynthesis has been reported very recently.^10^ Closer inspection of the biosynthesis products revealed that the octa-CDP producing hybrid synthetases EnSYN–Ba(T)C_3_ and BeSYN–Ba(T)C_3_ still produced trace amounts of the natural smaller ring size (Fig. 3a and c). Notably, the wild-type BaSYN is also able to produce minor amounts of hexa-bassianolide (Fig. 5b). Therefore, we can conclude that the BaSYN-C_3_ domain also cyclizes the premature CDP chain, which is in contrast to recent statements.^10^ The four BaSYN-based hybrid constructs BaSYN–En(T)C_3_ and BaSYN–Be(T)C_3_ produced the respective hybrid CDP hexa-bassianolide (corresponding to enniatin C, 3× *N*-Me-l-Leu, *m*/*z* 682.463 [M + H]^+^; Fig. 3b). Apart from BaSYN, hexa-bassianolide is also a natural side product of the wild-type EnSYN.^27^ The results reinforce the control function of the C_3_ domain with respect to the encrypted chain length for macrocyclization as well as its substrate flexibility concerning aliphatic and aromatic *N*-Me-l-AAs.

### A detailed view of the C_3_ domain

**Fig. 4:**
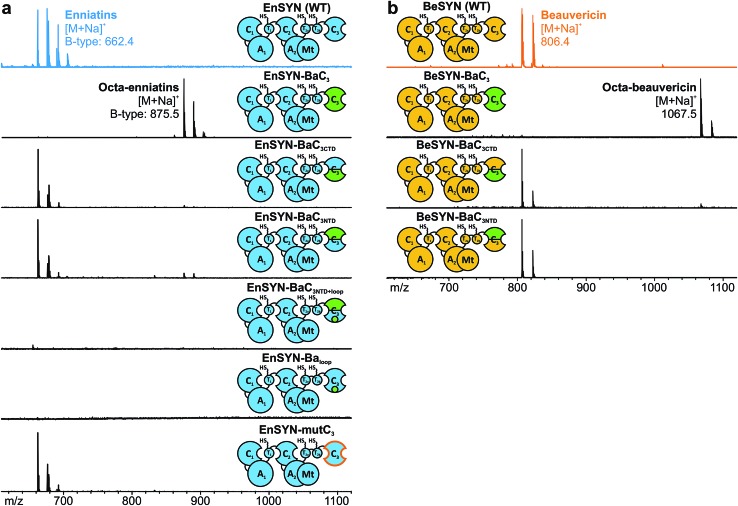
Swapping of C_3_ subdomains and elements thereof. *In vivo* CDP production of wild-type and hybrid synthetases based on EnSYN (a) and BeSYN (b) with swapped C_3NTD_, C_3CTD_, bridging loop region (small green circle) and five distinct AAs of BaSYN (orange line) in *E. coli*, respectively, as monitored by MALDI-TOF-MS.

In analogy to conventional elongation C domains (C_elong_) and fungal C_term_ domains,^20,22,28^ the C_3_ domain consists of an N- and C-terminal subdomain (C_NTD_, C_CTD_), which together form a V-shaped pseudo-dimer with the active site buried in the cleft between the two subdomains (ESI Fig. 3†). Whereas C_NTD_ harbors the catalytically relevant His_cat_, C_CTD_ provides most of the interface residues towards the donor T domain. In contrast to the C_term_ domain of the fungal tryptoquialanine (TqaA) synthetase that is closed at its acceptor site,^22^ the C_3_ domain of fungal CDP synthetases also catalyzes condensation reactions.^10^ Hence, the C_3_ domain features a functional acceptor site competent to catalyze ester bond formation. In order to gain insights into the molecular determinants of macrocyclization and ring size control by the C_3_ domain, we started with a sequence-based shuffling approach. An alignment of the C_3_ domains revealed ten residues potentially involved in ring size determination (ESI Fig. 5†). When visualized in a superposition of the TqaA-C_term_ structure^22^ with a structural model of EnSYN-C_3_ (ESI Fig. 9†), five from the originally ten conspicuous residues remained, which were swapped from the BaSYN to the EnSYN sequence (EnSYN-mutC_3_). Exchanging these five amino acids did not hamper CDP production, but did not lead to an altered ring size either (Fig. 4a), ruling out these residues in terms of ring size control.

In an alternative approach, we swapped the subdomains C_3NTD_ and C_3CTD_ of BaSYN into the EnSYN and BeSYN system (ESI Fig. 4†). Expression of the four constructs EnSYN–BaC_3NTD_, EnSYN–BaC_3CTD_, BeSYN–BaC_3NTD_ and BeSYN–BaC_3CTD_ in *E. coli* co-expressing NpgA showed that CDP production was not hampered by the extensive heterologous intra-domain interfaces (Fig. 4). Remarkably, a swap of C_3CTD_ gave rise to minor production of octa-CDPs. In the EnSYN-based system, a swap of C_3NTD_ even slightly enhanced production of octa-enniatin B (Fig. 4a), whereas only trace amounts of octa-beauvericin could be observed for BeSYN–BaC_3NTD_ (Fig. 4b). This data encouraged an additional swap of the bridging loop in C_3CTD_ (EnSYN–BaC_3NTD+loop_, EnSYN–Ba_loop_), the region which crosses over to C_3NTD_ and forms a lid above the cyclization pocket (ESI Fig. 3†). However, swapping of the bridging loop completely abolished CDP production (Fig. 4a), suggesting that the conformation of the C_3_ domain has been massively disturbed. Thus, we hypothesize that CDP ring size is controlled not only by the size of the substrate pocket, but also by the acceptor- and donor-site interfaces that mediate the recruitment and correct positioning of the corresponding partner T domains. Such regulation *via* the T–C interfaces becomes in particular relevant in light of the recently postulated linear mode of CDP synthetases,^10^ which relies on a competition between T_1_ and T_2a/b_ for binding to C_3_ leading either to chain elongation or macrocyclization.

### Scale-up production in *A. niger* and bioactivities of hybrid CDPs

In previous engineering approaches, the use of *E. coli* as a bacterial heterologous host proved valuable for rapid proof-of-principle approaches.^17^ However, the fungal heterologous host *A. niger* appears to be more suitable for scale-up of fungal CDP production, as it renders significantly higher CDP yields.^17,19^ Therefore, we aimed for an enhanced production of the hybrid CDPs octa-enniatin B, octa-beauvericin and hexa-bassianolide by using *A. niger* under control of an inducible expression system.^19^ For that purpose, we performed polyethylene glycol (PEG)-mediated transformation of two *A. niger* strains differing in their protease profiles (strains AB1.13 and MA169.4) with the hybrid synthetase genes of EnSYN–BaTC_3_, BeSYN–BaTC_3_, BaSYN–EnTC_3_ and BaSYN–BeTC_3_. The genes were integrated into the fungal genome by homologous recombination (ESI Fig. 10†). From the two alternative constructs BaSYN–EnTC_3_ and BaSYN–BeTC_3_ synthesizing hexa-bassianolide, the former was chosen for scale-up in *A. niger* due to a higher production (ESI Fig. 11†).

**Fig. 5:**
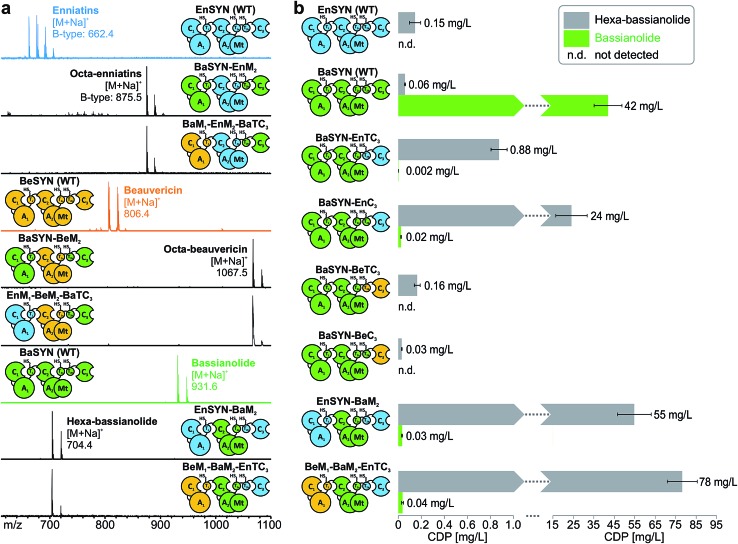
Multiple swapping of fungal CDP synthetases. (a) *In vivo* CDP production of the hybrid synthetases (see color code) monitored by MALDI-TOF-MS. (b) CDP titers of wild-type and hybrid NRPSs producing bassianolide and hexa-bassianolide in *E. coli* compared by LC-ESI-MS (*n* = 3 cultures, standard deviation). n. d.: not detected.

All hybrid CDPs were purified by extraction with ethyl acetate, flash chromatography and reversed phase HPLC. Purification of the hybrid octa-CDPs from *A. niger* yielded 4 mg L^–1^ of octa-enniatin B and 10.8 mg L^–1^ of octa-beauvericin, respectively. Intriguingly, purification of hexa-bassianolide produced by BaSYN–EnTC_3_ afforded very high titers of approximately 1.3 g L^–1^. To our knowledge, this is the first report of an artificial NRPS producing on the g L^–1^-scale, which underlines the great potential of fungal NRPS systems for biosynthetic engineering. Without understanding the catalytic assembly in detail, the higher titer in comparison to the hybrid octa-CDPs might be attributed to a more efficient interaction between the heterologous parts. For example, combining BaSYN with segments of EnSYN in *E. coli* rendered significantly higher titers than BaSYN–BeSYN combinations (Fig. 5b), which were also used for the production of octa-beauvericin (BeSYN–BaTC_3_). Since both wild-type BaSYN and EnSYN naturally produce minor amounts of hexa-bassianolide, the building blocks d-Hiv and l-Leu represent cognate substrates for both NRPS systems, thus ensuring efficient processing. To date, hexa-bassianolide could only be obtained in low amounts either from chemical synthesis,^29^ by precursor-directed biosynthesis (2.2 mg L^–1^)^30^ or from extraction of cultures of *Verticillium hemipterigenum* (BCC 1449; 0.3 mg L^–1^).^27^ In a very recent report on heterologous CDP production in *S. cerevisiae*, no yields were mentioned.^10^

Based on the sufficient amount of the hybrid CDPs for biological profiling and the broad activity range of CDPs,^30–32^ octa-enniatin B, octa-beauvericin and hexa-bassianolide were tested for antiparasitic, antibacterial and antifungal activity along with cytotoxicity measurements (ESI Tables 4–6†). As for hexa-bassianolide, antiplasmodial, antimycobacterial and cytotoxic properties were already shown.^30,31^ Additionally, hexa-bassianolide was found to be active against the parasites *Trypanosoma b. rhodesiense*, *Trypanosoma cruzi* and *Leishmania donovani* (ESI Table 4†). Most remarkably, both hybrid octa-CDPs showed a significantly increased antiparasitic activity (ESI Table 4†). Against *T. cruzi*, octa-enniatin B and octa-beauvericin showed up to 12-fold lower IC_50_ (1.76 μM and 0.54 μM, respectively) than the medical reference drug benznidazole (6.53 μM). Furthermore, they are superior to their corresponding natural hexa-CDPs enniatin B (2.31 μM) and beauvericin (0.76 μM), respectively. Against *L. donovani*, octa-enniatin B and octa-beauvericin showed up to >8-fold lower IC_50_ (0.07 μM and 0.14 μM, respectively) compared to the medically used reference drug miltefosine (0.58 μM). In comparison to enniatin B and beauvericin (IC_50_ 0.72 μM and 0.31 μM, respectively), both compounds were also significantly more active and showed a similar cytotoxicity against rat-derived *L6* cells (ESI Table 4†).

### Multiple swapping of fungal CDP synthetases

With the assigned synthetase functions for precursor and ring size determination in hand, the fungal iterative NRPSs can be seen as a modular assembly platform for a tailored construction of new CDP structures. In order to test the flexibility of fungal CDP synthetases even further, we assessed the influence of multiple domain swaps on hybrid CDP synthesis. For this purpose, we constructed a set of synthetase chimeras for each of the three hybrid CDPs comprising parts from up to three CDP synthetases. All of these six multiple hybrids (EnSYN–BaM_2_, BaSYN–EnM_2_, BaSYN–BeM_2_, BeM_1_–BaM_2_–EnTC_3_, EnM_1_–BeM_2_–BaTC_3_, BeM_1_–EnM_2_–BaTC_3_) were capable of producing the predicted hexa- or octa-CDPs in *E. coli* co-expressing NpgA (Fig. 5a). These results demonstrate the first successful multiple cross-pathway assembly in fungal iterative NRPSs.

Since bacterial NRPS-systems do not tolerate this type of swapping well,^13^ we further examined whether fungal CDP synthetases behave in a similar fashion. For that purpose, we determined the production titers of all wild-type and hybrid CDP synthetases producing hexa-bassianolide. The titers of all singly swapped hybrids (0.03–0.88 mg L^–1^) remained in the same range as that of wild-type EnSYN (0.15 mg L^–1^) and BaSYN (0.06 mg L^–1^), with the exception of BaSYN–EnC_3_ (24 mg L^–1^). The fact that BaSYN–EnC_3_ showed such a superior production in comparison to BaSYN–EnTC_3_ (0.88 mg L^–1^) highlights the influence of artificial T–C domain pairings in hybrid synthetases, which have a crucial role, *i.e.* in ester bond formation and macrocyclization. Surprisingly, the hybrid synthetases containing multiple swaps (EnSYN–BaM_2_, BeM_1_–BaM_2_–EnTC_3_) showed an enhanced production up to a factor of 520 (55–78 mg L^–1^) compared to the wild-type EnSYN (0.15 mg L^–1^), despite having more heterologous interfaces for domain–domain interactions. This is in stark contrast to virtually all previous studies on module and domain exchanges in bacterial NRPS systems suffering from a significant drop in product titers.^32^ Notably, the relatively high titer was achieved in the bacterial heterologous host *E. coli*, which previously rendered low production yields.^17^ Fungal NRPS systems might thus represent a viable alternative as an engineering platform with promising potential. Furthermore, the results suggest that the efficiency of the biosynthesis is influenced by the N-terminal part of the synthetase. This is supported by the fact that the hybrid EnSYN–BaM_2_ contains a homologous module 1 and T_2b_C_3_, thus pointing to highly important inter-modular interactions like the proposed C_3_–T_1_ interaction during ester bond formation.^10^ However, the highest production was achieved with a heterologous C_3_–T_1_ interface (BeM_1_–BaM_2_–EnTC_3_). The assumption of a readily exchangeable “bio-brick” system thus appears somewhat simplistic for a sustainable engineering approach.

## Conclusions

Fungal CDP synthetases produce six- and eight-membered CDPs with a broad range of pharmacologically relevant bioactivities. Based on their modular structure, high homology and manageable size, these nonribosomal assembly lines bear good prospects to be an effective engineering platform for the production of novel CDP compounds. Still, a tailored engineering of CDP synthetases is somewhat limited by our poor understanding of the biosynthesis mechanisms. The systematic investigation of the individual contributions of domains to the catalytic activity and synthesis products in this study allow us to confirm the following model: the C-terminal C_3_ domain performs macrocyclization and determines the ring size independently from C_1_. In contrast to recent findings,^10^ truncated synthetases lacking the C_1_ domain are indeed functional *in vivo*. However, since the absence of C_1_ significantly affects the production yields of CDPs (drop of synthesis to 1–55%), a stabilizing and/or supportive role of C_1_ in the catalytic cycle is apparent. In accordance with a recent crystal structure comprising the C–A–T–Te domains of the bacterial NRPS AB3403,^28^ the C_1_ domain may serve as a resting site during precursor adenylation in the A_1_ domain. This stabilization may also apply to the A_1_ domain itself, since the C domains can affect the tertiary structure^33^ and substrate specificities of their downstream A domain partners.^34^

With regard to substrate specificity, various functional hybrid synthetases with a heterologous C_3_ domain demonstrated that the C_3_ domain tolerates aliphatic as well as aromatic side chains (*N*-Me-l-Val/Leu/Ile/Phe). Apart from esterification, C_3_ also inherits the role of a gauge, measuring product chain length. In contrast to recent statements,^10^ this gauge also allows for cyclization of premature depsipeptide chains. Deletion of the Mt domain, affording the corresponding non-methylated peptides, was able to demonstrate that the absence of structure-modulating *N*-methylations is tolerated. As previous Mt domain deletions reportedly only affected β-methylation of Glu in daptomycin,^13^ our findings represent a rare example of complete *in vivo* NRP backbone engineering. The construction of a number of artificial CDP synthetases by means of domain swapping yielded hybrid hexa- and octa-CDPs under *in vivo* conditions. This way, we were able to produce the hybrid CDP hexa-bassianolide at very high titer of 1.3 g L^–1^ as well as new-to-nature octa-enniatin B (4 mg L^–1^) and octa-beauvericin (10.8 mg L^–1^) by using *A. niger* as a robust and cognate heterologous host. While previous approaches with the daptomycin synthetase have shown that multiple domain swaps lead to reduced product yields,^13^ we could significantly enhance CDP production *in vivo* by constructing functional hybrid synthetases from three different NRPSs. The results highlight the potential of fungal NRPS systems as engineering platforms as well as the crucial role of domain–domain and module–module interfaces in NRPS efficiency.

Swapping of parts of the C_3_ subdomain uncovered functional aspects of macrocyclization and ring size control. According to the bacterial Te mechanism, macrocyclization by the fungal C_3_ domain is performed when the free-standing hydroxy group at the tail of the linear depsipeptidyl chain is in close proximity to His_cat_ of C_3_ and the substrate thioester. In consequence, CDP ring size probably depends on the size and composition of the cyclization pocket formed by C_3NTD_ and C_3CTD_, along with a competitive process between the elongation and cyclization reaction. This assumption is supported by our observations that, unlike the octa-CDP-producing synthetases, all hexa-CDP-generating hybrids almost completely lost the capability of producing the cognate eight-membered ring. One reason might be the steric demand of a linear octa-depsipeptide, which may not fit well into the cyclization pocket of a hexa-CDP-producing synthetase. In addition to steric restrictions imposed by the (heterologous) cyclization pocket, ring size may also depend on intrinsic conformational properties of the growing depsipeptide chain. The nature of side chains, and in particular of backbone *N*-methylation, dictates the available conformational space of the depsipeptide backbone and thus affects cyclization efficiencies. For example, Ramachandran plots for peptides comprising *N*-Me-AAs demonstrate the restricted conformational freedom compared to their non-methylated analogs.^35^ However, the distinct mechanism of CDP ring size determination remains to be elucidated. By confirming recent findings of CDP production in synthetase variants lacking Ppant_2a/b_, our data also give strong support to the so-called “linear” CDP biosynthesis model. From a formal aspect, employing an alternating upstream and downstream elongation, this NRPS type might be reclassified as belonging to the “complex-type”^32^ rather than simply linear or iterative. We therefore suggest the term “looping mode”, implicating the substrate shuttling between two C domains in a consecutive manner. Still, since the role of the T_2b_ domain remains unclear and odd-numbered T domain-bound intermediates are yet to be observed,^8^ neither of the two models can be completely ruled out. Hence, further information must be gathered with synthetase mutants, *e.g.* stalled at certain biosynthesis steps, as it has been applied to the bacterial balhimycin synthetase^36^ and rifamycin polyketide synthase.^37^

Finally, our experiments show that the design of bio-combinatorial swaps in NRPSs can serve well for the synthesis of compounds with enhanced bioactivities. Accordingly, the bioactivity profiling of octa-enniatin B and octa-beauvericin rendered a significantly stronger antiparasitic activity against *T. cruzi* (up to 12-fold) and *L. donovani* (up to 8.3-fold) compared to the reference drugs benznidazole and miltefosine, respectively, accompanied by similar cytotoxicity against *L6* cells. Our findings give distinct clues for the active and applicable reprogramming of both iterative and linear NRPS assembly lines in further protein engineering approaches. In the future, we aim to expand the existing range of CDP ring sizes beyond the natural hexa- and octa-CDPs, giving rise to a whole class of new-to-nature cyclic fungal products with superior pharmacological activities and efficiencies.

## Conflicts of interest

There are no conflicts of interest to declare.
