# Supplementary material for: Harnessing fungal nonribosomal cyclodepsipeptide synthetases for mechanistic insights and tailored engineering
Source: Chem Sci. 2017 Sep 25;8(11):7834–43. doi: 10.1039/c7sc03093b (PMC5674221; doi:10.1039/c7sc03093b)
Supplement: Supplementary file 1 [file SC-008-C7SC03093B-s001.pdf]

Electronic Supplementary Material (ESI) for Chemical Science.
This journal is © The Royal Society of Chemistry 2017

Supporting Information

Harnessing Fungal Nonribosomal Cyclodepsipeptide Synthetases for Mechanistic Insights and Tailored Engineering

Charlotte Steiniger,[a] Sylvester Hoffmann,[a] Andi Mainz,[a] Marcel Kaiser,[b,c] Kerstin Voigt,[d] Vera Meyer,[e] and Roderich D. Süssmuth*[a]

Table of Contents

1. Supplementary Methods 2
2. Supplementary Tables 6
3. Supplementary Figures 27
4. References 48

1. Supplementary Methods

Strain Construction. Synthetase fragments and plasmid backbones (pGEX4T1, pET28a, pVG2.2) including 20 bp overhangs were amplified by PCR. The wild-type synthetases EnSYN, BeSYN and BaSYN were used as PCR template, the corresponding primers are given in Supplementary Table 7. For swapping of the bridging loop, the $ESYNC_{3CTD}$-part containing the swap was synthesized by Thermo Fisher (Darmstadt, Germany). The ESYNC3 domain containing the five site-directed mutations was also synthesized by Thermo Fisher (Darmstadt, Germany). Both were used as a PCR-template. Mutation of the two Ser-residues carrying the $Ppant_{2a/b}$ was performed with mutational primers (Supplementary Table 7). PCR reactions, Gibson cloning and transformation of *E. coli* DH5α (K12) and partial sequencing of the N- and C-terminal synthetase ends and swapping/truncation/mutation sites were followed according to standard protocols (Supplementary Table 11,12; Supplementary Fig. 12, 13).[1,2] Using the transformation procedure stated above, verified plasmids (0.5 µL) were retransformed into the expression strain *E. coli* BL21 gold (DE3), harbouring an additional PPTase on a second plasmid derived from *A. nidulans* DM3365 (pACYCduet_npgA). For comparison of the different PPTases, pGEX4T1_ESYN-$BaTC_3$ was additionally transformed into *E. coli* BL21 gold (DE3) co-expressing Sfp and E. *coli* BL21 gold (DE3) co-expressing Svp. For the scale-up production of octa-enniatin B, octa-beauvericin and hexa-bassianolide, the four hybrids EnSYN-$BaTC_3$, BeSYN-$BaTC_3$, BaSYN-$EnTC_3$ and BaSYN-$BeTC_3$ were cloned into the *A. niger* transformation vector pVG2.2. PEG-mediated transformation was carried out using *A. niger* AB1.13 and MA169.4 as uracil-auxotroph recipient strains and single copy transformants were isolated, purified and verified by PCR (amplification of A2 domain region) according to standard protocols.[3]

Cultivation conditions. *E. coli* BL21 gold-npgA producing GST-tagged wild-type and hybrid synthetases was expressed in dYT auto-induction medium (30 mL) containing ampicillin (100 µg/mL) and chloramphenicol (34 µg/mL). Cultivation was carried out at 37 °C and 200 rpm for 3 h, before cultures were supplemented with D-Hiv (10 mM) for hybrid CDP production. Further incubation was carried out at 18 °C and 200 rpm for 20 h. Harvested cells were frozen at -20 °C. For CDP production in *A. niger*, 1 L CM-medium supplemented with glucose (5 %) and talcum (1 %) was inoculated with $5x10^6$ spores/mL of selected transformants CS2.6 (octa-beauvericin), CS5.3 (octa-enniatin B) or CS6.15 (hexa-bassianolide) and cultivated for 16 h at 28 °C and 230 rpm. Synthetase expression was induced by addition of doxycycline (10 mM). CDP production was triggered by supplementation of the cultures with D-Hiv (10 mM) and the corresponding amino acid L-Val, L-Leu or L-Phe (20 mM) at 0 h, 24 h and 48 h. Mycelium was harvested by filtration after 72 h of cultivation.

SDS-PAGE synthetase analytics. Cell pellets obtained from *E. coli* were frozen for 1 h, thawed and lysed with a homogenizer. Following digestion of the homogenized cells with DNase, samples were centrifuged at 14,000 rpm, 15 min, 4 °C. Supernatant and pellet were separated and prepared for SDS-PAGE (Supplementary Fig. 8). Protein bands of the expected size were excised and digested with trypsin for 16 h according to the method of Shevchenko *et al.* (2007).[4] Digested synthetases were analyzed by LC-ESI-Orbitrap-IDA (Orbitrap XL mass spectrometer (Thermo Fisher, Dreieich, Germany; Column: Vydac 218MS C18 5u 150mm ID2.1 mm (GRACE, Worms, Germany); Flow rate: 0.3 mL/min; Solvent A: water (0.1 % HCOOH), Solvent B: acetonitrile (0.1 % HCOOH), IDA mode (TOP3), gradient Supplementary Table 14). Characteristic fingerprint peptides of the respective synthetase were compared to *in silico* digestion data of the *E. coli* BL21 (DE3) proteome supplemented with the respective hybrid synthetase sequence (MSConvert,[5] SearchGUI,[6] PeptidShaker;[7] Supplementary Table 20).

CDP metabolite analytics. Metabolites were extracted from cell pellets of *E. coli* or from *A. niger* mycelium (100 mg) according to a recently published protocol.[8] For CDP analysis by MALDI-TOF-MS, samples were taken up in isopropanol/water (50 µL; 1:1) and vortexed. Each sample was mixed with the matrix dihydroxybenzoic acid (saturated DHB; 2 µL; 1:1) and the mixture (1 µL) was spotted on a MALDI target plate. Measurements were performed on a MALDI-TOF(/TOF) ultrafleXtreme mass spectrometer (Bruker, Karlsruhe, Germany) in reflective-positive (RP) mode for MS and LIFT mode for MS/MS information (Supplementary Fig. 15). For CDP analysis by LC-ESI-MS (QQQ), samples were taken up in isopropanol/water (1 mL; 1:1), diluted 1:100 in isopropanol/water (1:1) and vortexed. Samples were centrifuged (14,000 rpm, 10 min, RT) and 700 µL were transferred to an HPLC vial. Measurements were performed on an ESI-Triple-Quadrupole mass spectrometer (6460 Series, Agilent Technologies, Waldbronn, Germany; UHPLC 1290 Infinity-Series (Agilent Technologies, Waldbronn, Germany); Column: Poroshell 120 EC-C18 3.0x50 mm (Agilent, Waldbronn, Germany); Flow rate: 0.4 mL/min. Solvent A: water, Solvent B: isopropanol; gradient Supplementary Table 15). MS/MS-spectra of desmethyl-enniatin and desmethyl-bassianolide were obtained in MS/MS-mode (Supplementary Table 16, Supplementary Fig. 14). Extracts from *E. coli* BL21 gold_EnSYN-BaTC$_3$ were measured in MRM-mode (Supplementary Table 16) in order to compare the PPTases NpgA derived from *A. nidulans* (DSM 3365), Sfp derived from *Bacillus subtilis ssp. spizizenii* (ATCC6633) and Svp derived from *Streptomyces mobaraensis* (DSM40903) (Supplementary Fig. 7). Production of hexa-bassianolide by the two hybrids BaSYN-EnTC$_3$ and BaSYN-BeTC$_3$ was also compared by LC-ESI-MRM (*n*=3 cultures with standard deviation, Supplementary Table 16) to select high-level *A. niger* expression clones for scale-up experments (strains CS2.6, CS5.3 and CS6.15; Supplementary Fig. 11).

Hybrid CDP metabolite isolation. To purify the hybrid CDPs from the *A. niger* strains CS2.6, CS5.3 and CS6.15, harvested mycelium from 1 L culture was lyophilized and extracted 4 times with ethyl acetate (4 h, 4 h, 14 h, 4 h). The crude extracts were purified in the first step by flash reversed phase chromatography on a Reveleris Flash System (GRACE, Worms, Germany; Column: Reveleris C18-WP flash cartridge, 40 g (GRACE, Worms, Germany); Flow rate: 40 mL/min; Solvent A: water (0.1 % HCOOH), Solvent B: acetonitrile (0.1 % HCOOH); gradient Supplementary Table 17). Collected fractions from flash chromatography were analyzed by LC-ESI-MS (Supplementary Table 13). CDP-containing fractions were pooled and purified further (Supplementary Fig. 16) on a preparative HPLC system (1100 series, Agilent Technologies, Waldbronn, Germany; Column: Grom-Sil 120 ODS-4 HE, 10 µm, 250 mm, ID:20 mm (GRACE Worms, Germany); Flow rate: 15 mL/min; Solvent A: water (0.1 % HCOOH), Solvent B: acetonitrile (0.1 % HCOOH); gradient Supplementary Table 18). Collected HPLC fractions were analyzed by LC-ESI-MS (Supplementary Table 13). Octa-enniatin B was additionally purified on an analytical HPLC system (1200 series, Agilent Technologies, Waldbronn, Germany; Column: Luna 5u C18(2), 100 A, 100x4.6 mm, 5 micron (Phenomenex, Torrance, CA, USA); Flow rate: 1.5 mL/min; Solvent A: water (0.1 % HCOOH), Solvent B: acetonitrile (0.1 % HCOOH); gradient Supplementary Table 19). CDP-containing fractions were pooled and analyzed for purity by LC-ESI-MS (Supplementary Table 13, Supplementary Fig. 17).

NMR-spectroscopy. The purified compounds octa-enniatin B, octa-beauvericin and hexa-bassianolide were dissolved in 600 µL chloroform-d_1 and filled into 5 mm tubes. NMR spectra were acquired on a Bruker Avance III 700 MHz spectrometer using a TXI inverse probe head (Karlsruhe, Germany). Acquisition, processing and analysis of NMR data (Supplementary Table 21-23, Supplementary Fig. 18) were performed with TopSpin 3.5 (Bruker, Karlsruhe, Germany). Data sets were recorded at an effective temperature of 299 K. 2D ^{1}H-^{1}H COSY spectra were recorded with acquisition times of 180 ms and 22 ms in the direct and indirect ^{1}H dimension, respectively (Supplementary Fig. 19, 22). ^{1}H-^{13}C HSQC spectra were recorded with acquisition times of 180 ms and 10 ms in the direct ^{1}H and indirect ^{13}C dimension, respectively (Supplementary Fig. 20, 23). A delay Δ/2 of 1.72 ms was used for INEPT transfers corresponding to $^1J_{HC}$ of 145 Hz. ^{1}H-^{13}C HMBC spectra were recorded with acquisition times of 180 ms and 7 ms in the direct ^{1}H and indirect ^{13}C dimension, respectively (Supplementary Fig. 21, 24). A delay of 50 ms was used to achieve evolution of long-range $^{2,3}J_{HC}$ couplings of about 10 Hz. Apodization of time domain data was performed using either a sine bell function (COSY, HMBC) or a squared sine bell function shifted by 90° (HSQC). The 2D data was processed by applying linear forward prediction and zero filling prior to Fourier transformation.

Bioactivity assays. Purified octa-enniatin B, octa-beauvericin and hexa-bassianolide were tested for antiparasitic, antibacterial and antifungal bioactivity together with standards of enniatin B, beauvericin and bassianolide. Antiparasitic activity assays were performed against *Trypanosoma brucei rhodesiense* STIB 900 (trypomastigotes), *Trypanosoma cruzi* Tulahuen C4 (amastigotes) and *Leishmania donovani* MHOM-ET-67/L82 (host free axenic amastigotes) to determine the IC_{50} values as previously described by Orhan *et al.* (2010) (Supplementary Table 4).[9] Additionally, cytotoxicity was determined with an *L6* rat-derived cell line (Supplementary Table 4). As controls, reference drugs melarsoprol (*T. b. rhodesiense*), benznidazole (*T. cruzi*), miltefosine (*L. donovani*) and podophyllotoxin (cytotox) were used. For antibacterial testing, the following strains were used: *Bacillus subtilis* STI:10880, *Psudomonas aeruginosa* ST:337721 (multi-resistent), *Staphylococcus aureus* ST:33793, *Enterococcus faecalis* ST:33700 (vancomycin-resistent) and *Mycobacterium vaccae* STI:10670 (Supplementary Table 5). Antifungal activity was determined against *Sporobolomyces salmonicolor* ST:35974, *Candida albicans* STI:25000, *Penicillium notatum* STI:50164 and *Aspergillus fumigatus* Afum:00073 = ATCC46645 (Supplementary Table 6). The bacterial and fungal test strains are deposited at the Jena Microbial Resource Collection at www.jmrc.hki-jena.de. Activity was determined based on inhibition zone diameters in comparison to the reference drugs ciprofloxacin (antibacterial) and amphotericin B (antifungal) in accordance to Krieg *et al.* (2017).[10]

2. Supplementary Tables

Supplementary Table 1: C-domain core motifs in the C_1 domain.

Domain	Core Motif	SYN	Consensus Sequence										
C_1	1*	**general**	**S**	**x**	**A**	**Q**	**x**	**R**	**L/M**	**W/Y**	**x**	**L**	
		Ba	P	C	T	P	F	Q	R	D	V	I	
		PF	P	C	T	S	F	Q	C	D	V	I	
		En	P	G	T	P	F	Q	R	D	V	I	
		Be	P	C	T	P	F	Q	Y	D	V	L	
	2	**general**	**R**	**H**	**E**	**x**	**L**	**R**	**T**	**-**	**x**	**F**	
		Ba	Y	T	P	A	L	R	T	C	I	F	
		PF	Q	T	P	I	L	R	T	G	I	F	
		En	H	T	P	A	L	R	T	C	T	F	
		Be	R	T	P	A	L	R	A	C	T	F	
	3	**general**	**M**	**H**	**H**	**x**	**I**	**S**	**D**	**G**	**W/V**	**S**	
		Ba	F	S	H	S	F	V	D	S	A	F	
		PF	F	S	H	A	L	V	D	Y	T	V	
		En	F	S	H	A	L	V	D	S	T	F	
		Be	F	H	L	A	L	V	D	S	T	V	
	4*	**general**	**Y**	**x**	**D**	**F/Y**	**A**	**V**	**W**				
		Ba											
		PF	not found										
		En											
		Be											
	5*	**general**	**I/V**	**G**	**x**	**F**	**V**	**N**	**T**	**Q/L**	**C/A**	**x**	**R**
		Ba	D	G	P	T	S	T	V	V	P	F	R
		PF	D	G	P	A	R	T	V	V	P	I	R
		En	D	G	P	T	R	T	V	V	P	I	R
		Be	N	G	P	T	R	S	V	V	P	F	R
	6*	**general**	**H/N**	**Q**	**D**	**Y/V**	**P**	**F**	**E**				
		Ba	F	A	H	A	G	L	C				
		PF	F	E	H	A	G	L	R				
		En	F	A	H	A	G	L	R				
		Be	F	A	H	V	G	L	C				
	7*	**general**	**R**	**D**	**x**	**S**	**R**	**N**	**P**	**L**			
		Ba											
		PF	not found										
		En											
		Be											

* Core motif could not be unambiguously assigned.

Supplementary Table 2: C-domain core motifs in the C_2 domain.

Domain	Core Motif	SYN	Consensus Sequence										
		general	S	x	A	Q	x	R	L/M	W/Y	x	L	
		Ba	S	Y	S	Q	G	R	L	W	F	L	
	1	PF	S	F	A	Q	G	R	L	W	F	L	
		En	S	Y	A	Q	N	R	M	W	F	L	
		Be	S	Y	S	Q	G	R	L	W	F	L	
		**general**	**R**	**H**	**E**	**x**	**L**	**R**	**T**	**-**	**x**	**F**	
		Ba	R	H	E	T	L	R	T	-	T	F	
	2	PF	R	H	E	T	L	R	T	-	T	F	
		En	R	H	E	T	L	R	T	-	T	F	
		Be	R	H	E	T	L	R	T	-	T	F	
		**general**	**M**	**H**	**H**	**x**	**I**	**S**	**D**	**G**	**W/V**	**S**	
		Ba	M	H	H	I	I	S	D	G	W	S	
	3	PF	M	H	H	I	I	S	D	G	W	S	
		En	M	H	H	I	I	S	D	G	W	S	
		Be	M	H	H	I	V	S	D	G	W	S	
		**general**	**Y**	**x**	**D**	**F/Y**	**A**	**V**	**W**				
		Ba	Y	R	D	F	S	V	W				
C_2	4	PF	Y	R	D	F	S	V	W				
		En	Y	S	D	F	A	V	W				
		Be	Y	R	D	F	S	M	W				
		**general**	**I/V**	**G**	**x**	**F**	**V**	**N**	**T**	**Q/L**	**C/A**	**x**	**R**
		Ba	I	G	F	F	V	N	T	Q	C	M	R
	5	PF	I	G	F	F	V	N	T	Q	C	M	R
		En	I	G	F	F	V	N	T	Q	C	M	R
		Be	I	G	F	F	V	N	T	Q	C	M	R
		**general**	**H/N**	**Q**	**D**	**Y/V**	**P**	**F**	**E**				
		Ba	H	E	D	V	P	F	E				
	6	PF	H	Q	D	V	P	F	E				
		En	H	E	D	V	P	F	E				
		Be	N	E	D	V	P	F	E				
		**general**	**R**	**D**	**x**	**S**	**R**	**N**	**P**	**L**			
		Ba	R	D	L	S	Q	N	P	L			
	7	PF	R	D	L	S	R	N	P	L			
		En	R	D	L	S	R	T	P	L			
		Be	R	D	L	S	Q	T	P	L			

Supplementary Table 3: C-domain core motifs in the C_3 domain.

Domain	Core Motif	SYN	Consensus Sequence										
C_3	1	**general**	**S**	**x**	**A**	**Q**	**x**	**R**	**L/M**	**W/Y**	**x**	**L**	
		Ba	L	A	T	H	L	Q	Q	C	F	L	
		PF	P	A	T	Q	M	Q	R	V	F	L	
		En	P	S	T	Q	M	Q	K	A	F	L	
		Be	P	A	T	H	M	Q	K	A	F	L	
	2	**general**	**R**	**H**	**E**	**x**	**L**	**R**	**T**	**-**	**x**	**F**	
		Ba	K	Y	D	I	F	R	T	-	I	F	
		PF	H	F	D	I	F	R	T	-	V	F	
		En	K	L	D	M	F	R	T	-	V	F	
		Be	R	F	D	M	F	R	T	-	V	V	
	3	**general**	**M**	**H**	**H**	**x**	**I**	**S**	**D**	**G**	**W/V**	**S**	
		Ba	L	S	H	A	L	Y	D	G	L	S	
		PF	L	S	H	A	L	Y	D	G	L	S	
		En	M	S	H	A	L	Y	D	G	L	S	
		Be	L	S	H	A	L	Y	D	G	L	S	
	4	**general**	**Y**	**x**	**D**	**F/Y**	**A**	**V**	**W**				
		Ba	A	T	Q	F	S	R	Y				
		PF	P	P	K	F	A	G	Y				
		En	P	T	Q	F	A	R	Y				
		Be	A	N	Q	F	S	R	Y				
	5	**general**	**I/V**	**G**	**x**	**F**	**V**	**N**	**T**	**Q/L**	**C/A**	**x**	**R**
		Ba	I	G	P	C	T	N	A	V	P	V	R
		PF	I	G	P	C	L	N	Q	V	P	V	R
		En	I	G	P	C	T	N	A	V	P	V	H
		Be	V	G	P	C	T	N	A	V	P	V	R
	6	**general**	**H/N**	**Q**	**D**	**Y/V**	**P**	**F**	**E**				
		Ba	Y	E	T	I	G	F	D				
		PF	F	E	T	L	G	Y	D				
		En	F	E	S	L	G	F	E				
		Be	F	E	T	L	D	F	D				
	7*	**general**	**R**	**D**	**x**	**S**	**R**	**N**	**P**	**L**			
		Ba	D	W	P	D	S	A	R	N			
		PF	D	W	P	D	V	P	A	T			
		En	D	W	P	E	E	L	T	N			
		Be	N	W	P	A	T	A	N	N			

* Core motif could not be unambiguously assigned.

Supplementary Table 4: Antiparasitic activity and cytotoxicity of natural and hybrid CDPs. Antiparasitic activity was analyzed against *Trypanosoma brucei rhodesiense* STIB 900 (trypomastigotes), *Trypanosoma cruzi* Tulahuen C4 (amastigotes) and *Leishmania donovani* MHOM-ET-67/L82 (host free axenic amastigotes) as IC_{50}-values. Cytotoxicity was analyzed with rat-derived *L6* cells.

		IC_{50} [µM]			
	Compound	*Trypanosoma b. rhodesiense*	*Trypanosoma cruzi*	*Leishmania donovani*	Cytotox (*L6*)
Control drug	Melarsoprol	0.007			28.6
	Benznidazole		6.532		>380
	Miltefosine			0.576	138
	Podophyllotoxin				0.031
Wild-type CDP	Enniatin B	1.047	2.305	0.719	4.110
	Beauvericin	1.633	0.764	0.311	1.939
	Bassianolide	1.694	1.391	0.195	5.114
Hybrid CDP	Enniatin C	1.260	3.520	0.315	6.350
	Octa-enniatin B	0.873	1.752	0.070	3.903
	Octa-beauvericin	0.782	0.533	0.144	1.579

Supplementary Table 5: Antibacterial activity of natural and hybrid CDPs. Activity was analyzed by comparison of inhibition zone diameters.

		Bacillus subtilis	*Pseudomonas aeruginosa*	*Staphylococcus aureus* (multi-resistent)	*Enterococcus faecalis* (vancomycin-resistent)	*Mycobacterium vaccae*
	JMRC-No.	STI:10880	ST:337721	ST:33793	ST:33700	STI:10670
Control drug	Ciprofloxacin	29	28/38p	0	16F	20p
	Solvent	0	10	0	0	0
Wild-type CDP	Beauvericin	18	14P	13	18F	27
	Bassianolide	0	0	0	0	12p
	Enniatin B	15p	0/A	15P	17p(F)	24
Hybrid CDP	Enniatin C	10	0/A	0	0	14p
	Octa-beauvericin	0	0	0	0	12p

p- Colonies in inhibition zone
P- Many colonies in inhibition zone
F- Promotion of inhibition zone
A- Intimation of inhibition

Supplementary Table 6: Antifungal activity of natural and hybrid CDPs. Activity was analyzed by comparison of inhibition zone diameters.

	Sporobolomyces salmonicolor	***Candida albicans***	***Penicillium notatum***	***Aspergillus fumigatus***
JMRC-No.	ST:35974	STI:25000	STI:50164	ATCC 46645
Beauvericin	0	0	14p	n.d.
Bassianolide	0	0	0	0
Enniatin B	0	0	13/18p/25P	0
Enniatin C	0	0	0	0
Octa-beauvericin	0	0	0	n.d.
Amphotericin B	18p	20	18p	20
Solvent	10	0	10	0

p- Colonies in inhibition zone
P- Many colonies in inhibition zone

Supplementary Table 7: Primers used in this study.

Primer	Sequence 5' → 3'	Usage
E. coli		
pGEX4T1_for	GCATCGTGACTGACTGACGATCTG	pGEX4T1 backbone
pGEX4T1_rev	GGCACGCGGAACCAGATCCGATTTTGGAG	pGEX4T1 backbone
En-pET28a_for	GTTTGAACGAGGCTTTGTAGGCACCACCACCACCACCACTG	pET28a backbone
En-pET28a_rev	GATATCGTCGCCAGACTCCTCGTAGCTGCCCTGGAAATACAAG	pET28a backbone
Ba-pET28a_for	GCTTTGAATGCGTCTTTATGAGCACCACCACCACCACCACTG	pET28a backbone
Ba-pET28a_rev	GGTACCCCCGCTGGAGCTGCCCTGGAAATACAAGTTTTC	pET28a backbone
pGEX-EnM1_for	CGGATCTGGTTCCGCGTGCCATGTCACTCCACACCCCAAG	EnSYN<br>EnSYN-BaTC$_3$<br>EnSYN-BaC$_3$<br>EnSYN-BaC$_{3CTD}$<br>EnSYN-BaC$_{3NTD}$<br>EnSYN-BaC$_{3NTD+loop}$<br>EnSYN-Ba$_{loop}$<br>EnSYN-mutC$_3$<br>EnSYNΔMt<br>EnSYNΔC$_3$<br>EnSYN-BaM$_2$<br>EnSYNΔPpant$_{2a}$<br>EnSYNΔPpant$_{2b}$<br>EM$_1$-BeM$_2$-BaTC$_3$
pGEX-BeauM1_for	CGGATCTGGTTCCGCGTGCCATGGAGCCGCTCAAAAATG	BeSYN<br>BeSYN-BaTC$_3$<br>BeSYN-BaC$_3$<br>BeM$_1$-BaM$_2$-EnTC$_3$<br>BeSYN-BaC$_{3CTD}$<br>BeSYN-BaC$_{3NTD}$<br>BeM$_1$-EnM$_2$-BaTC$_3$
En-BassT3_for	GGACTTGCTATGCAGAACACCCTCCTCCCTACAGCTTC	EnSYN-BaTC$_3$<br>BaSYN-EnM$_2$<br>BeM$_1$-EnM$_2$-BaTC$_3$
Be-BassT3_for	GGGGCTGCAAAACGTCGTGACCCTCCTCCCTACAGCTTC	BeSYN-BaTC$_3$<br>EnM$_1$-BeM$_2$-BaTC$_3$<br>BaSYN-BeM$_2$
pGEX-BassC3_rev	CGTCAGTCAGTCACGATGCTCATAAAGACGCATTCAAAG	BaSYN<br>EnSYN-BaC$_3$<br>EnSYN-BaTC$_3$<br>BeSYN-BaTC$_3$<br>BeSYN-BaC$_3$<br>EnSYN-BaC$_{3CTD}$<br>BaSYNΔC$_1$<br>BaSYNΔMt<br>BaSYN-EnM$_2$<br>BaSYN-BeM$_2$<br>BeSYN-BaC$_{3CTD}$<br>BeM$_1$-EnM$_2$-BaTC$_3$<br>EnM$_1$-BeM$_2$-BaTC$_3$
Ba-EnM2_rev	GAAGCTGTAGGGAGGAGGGTGTTCTGCATAGCAAGTCC	EnSYN-BaTC$_3$<br>BaSYN-EnM$_2$<br>BeM$_1$-EnM$_2$-BaTC$_3$
Ba-BeauM2_rev	GAAGCTGTAGGGAGGAGGGTCACGACGTTTTGCAGCCCC	BeSYN-BaTC$_3$<br>EnM$_1$-BeM$_2$-BaTC$_3$<br>BaSYN-BeM$_2$
pGEX-BassM1_for	GGATCTGGTTCCGCGTGCCATGGAGCCACCCAACAAC	BaSYN<br>BaSYN-EnC$_3$<br>BaSYN-EnTC$_3$

Primer	Sequence 5' → 3'	Usage
		BaSYN-BeTC$_3$<br>BaSYN-BeC$_3$<br>BaSYNΔMt<br>BaSYN-EnM$_2$<br>BaSYN-BeM$_2$
En-BassM2_rev	CCTGTCCTTCAGCAACAGGATTCTTCGAGGCCAACTG	BaSYN-EnTC$_3$<br>BeM$_1$-BaM$_2$-EnTC$_3$<br>EnSYN-BaM$_2$
Ba-EnT3_for	CAGTTGGCCTCGAAGAATCCTGTTGCTGAAGGACAGG	BaSYN-EnTC$_3$<br>EnSYN-BaM$_2$
pGEX-EnC3_rev	CAGTCAGTCACGATGCGGCCCTACAAAGCCTCGTTCAAAC	EnSYN<br>BaSYN-EnC$_3$<br>BaSYN-EnTC$_3$<br>EnSYN-BaC$_{3NTD}$<br>EnSYN-BaC$_{3NTD+loop}$<br>EnSYNΔC$_1$<br>EnSYN-mutC$_3$<br>EnSYNΔMt<br>EnSYN-Ba$_{loop}$<br>EnSYN-BaM$_2$<br>EnSYNΔPpant$_{2a}$<br>EnSYNΔPpant$_{2b}$<br>BeM$_1$-BaM$_2$-EnTC$_3$
Be-BassM2_rev	CCACCATTCAAAGCCACGGGATTCTTCGAGGCCAACTG	BaSYN-BeTC$_3$
Ba-BeauT3_for	CAGTTGGCCTCGAAGAATCCCGTGGCTTTGAATGGTGG	BaSYN-BeTC$_3$
pGEX-BeauC3_rev	CAGTCAGTCACGATGCGGCCTCACAAAGCCGAGTTTAGAC	BeSYN<br>BaSYN-BeTC$_3$<br>BaSYN-BeC$_3$<br>BeSYNΔC$_1$<br>BeSYN-BaC$_{3NTD}$
Ba-EnT3_rev	CGGTACCCCCGCTGGAGCTCTTGGAATGCGAAGACTCC	EnSYN-BaC$_3$<br>EnSYN-BaC$_{3NTD}$<br>EnSYN-BaC$_{3NTD+loop}$
En-BassC3_for	GGAGTCTTCGCATTCCAAGAGCTCCAGCGGGGGTACCG	EnSYN-BaC$_3$<br>EnSYN-BaC$_{3NTD}$<br>EnSYN-BaC$_{3NTD+loop}$
En-BassT3_rev	CGCCAGACTCCTCGTAGCTTTCCAATTGAGAAACCTCTAG	BaSYN-EnC$_3$
Ba-EnC3_for	CTAGAGGTTTCTCAATTGGAAAGCTACGAGGAGTCTGGCG	BaSYN-EnC$_3$
Ba-BeauC3_for	GAGGTTTCTCAATTGGAAAGCGACAGAGTAAAGCACAC	BaSYN-BeC$_3$
Be-BassT3_rev	GTGTGCTTTACTCTGTCGCTTTCCAATTGAGAAACCTC	BaSYN-BeC$_3$
Be-BassC3_for	GAGCTGGGTCAGTTGGAGAGCTCCAGCGGGGGTACCG	BeSYN-BaC$_3$<br>BeSYN-BaC$_{3NTD}$
Ba-BeauT3_rev	CGGTACCCCCGCTGGAGCTCTCCAACTGACCCAGCTC	BeSYN-BaC$_3$<br>BeSYN-BaC$_{3NTD}$
En-BaC3ctd_for	GTATGCTGCACACAGTCGTGGACCTGGTTGCGACTTTTGG	EnSYN-BaC$_{3CTD}$
Ba-EnC3ntd_rev	CCAAAAGTCGCAACCAGGTCCACGACTGTGTGCAGCATAC	EnSYN-BaC$_{3CTD}$
Bass-EnC3ctd_for	GTACGTGGACCATACTCGAGAAGAAGGTTATCCCTTCTG	EnSYN-BaC$_{3NTD}$<br>EnSYN-BaC$_{3NTD+loop}$
En-BassC3ntd_rev	CAGAAGGGATAACCTTCTTCTCGAGTATGGTCCACGTAC	EnSYN-BaC$_{3NTD}$<br>EnSYN-BaC$_{3NTD+loop}$
En_EnC3ctd_for	GTATGCTGCACACAGTCGTGAAGAAGGTTATCCCTTCTGG	EnSYN-Ba$_{loop}$
En_EnC3ntd_rev	CCAGAAGGGATAACCTTCTTCACGACTGTGTGCAGCATAC	EnSYN-Ba$_{loop}$
pGEX-EA1_for	GGATCTGGTTCCGCGTGCCGTGGAAAAGGTGGACATG	EnSYNΔC$_1$<br>EnSYNΔC$_1$C$_3$
pGEX-BassA1_for	GGATCTGGTTCCGCGTGCCGTGGGACAGCTGGATGTTCTG	BaSYNΔC$_1$
pGEX-BeauA1_for	GGATCTGGTTCCGCGTGCCGTGAAACAACTAGACATTGTG	BeSYNΔC$_1$

**Primer**	**Sequence 5' $\rightarrow$ 3'**	**Usage**
En-EnC3_for	AGCTACGAGGAGTCTGGCGACGATATCCAG	EnSYN-mutC$_3$
En-EnT3_rev	CTGGATATCGTCGCCAGACTCCTCGTAGC	EnSYN-mutC$_3$
EnwoMt_b_for	CGGATTCATCGTCGCGGACGCCGCTCTGCAAGTCCG	EnSYNΔMt
EnwoMt_a_rev	CGGACTTGCAGAGCGGCGTCCGCGACGATGAATCCGACC	EnSYNΔMt
BasswoMt_a_rev	CATTCACGAACCTGCACCGCCACGTCGTGTTCCGCAACCAC	BaSYNΔMt
BasswoMt_b_for	GTGGTTGCGGAACACGACGTGGCGGTGCAGGTTCGTGAATG	BaSYNΔMt
pGEX-EnT3_rev	CGTCAGTCAGTCACGATGCTCACTTGGAATGCGAAGACTC	EnSYNΔC$_3$<br>EnSYNΔC$_1$C$_3$
pET-EnC3_rev	CAGTGGTGGTGGTGGTGGTGCCTACAAAGCCTCGTTCAAAC	EnSYN-C$_3$
pET-EnC3_for	CTTGTATTTCCAGGGCAGCTACGAGGAGTCTGGCGACGATATC	EnSYN-C$_3$
BaC3-pET_rev	GTGGTGGTGGTGGTGGTGCTCATAAAGACGCATTCAAAGC	BaSYN-C$_3$
pET-BaC3_for	GAAAACTTGTATTTCCAGGGCAGCTCCAGCGGGGGTACC	BaSYN-C$_3$
En-BassM2_for	GTCATTGGTACTTCGCTGACCGTCACCAGCATCCCG	EnSYN-BaM$_2$
Ba-EnM1_rev	GCCGGGATGCTGGTGACGGTCAGCGAAGTACCAATGAC	EnSYN-BaM$_2$
Ba-EnM2_for	CTATTGGTGGCAGCTCAATGCCATACAGCCTTATTCCC	BaSYN-EnM$_2$
En-BassM1_rev	GGGAATAAGGCTGTATGGCATTGAGCTGCCACCAATAG	BaSYN-EnM$_2$
En-T2aStoA_for	CTTCCAGCTCGGCGGTCACGCTCTCCTCGCTACGAAAC	EnSYNΔPpant$_{2a}$
En-T2aStoA_rev	GTTTCGTAGCGAGGAGAGCGTGACCGCCGAGCTGGAAG	EnSYNΔPpant$_{2a}$
En-T2bStoA_for	CGATCTCGGTGGTCACGCGCTCATGGCTACTAAGC	EnSYNΔPpant$_{2b}$
En-T2bStoA_rev	GCTTAGTAGCCATGAGCGCGTGACCACCGAGATC	EnSYNΔPpant$_{2b}$
Be-BassM2_for	CGCCCTCGACCATCACGACGACCGTCACCAGCATCC	BeM$_1$-BaM$_2$-EnTC$_3$
Ba-BeauvM1_rev	GGATGCTGGTGACGGTCGTCGTGATGGTCGAGGG	BeM$_1$-BaM$_2$-EnTC$_3$
En-BeauvM2_for	GTCATTGGTACTTCGCTGCCCTTTGCCACCATTCC	EnM$_1$-BeM$_2$-BaTC$_3$
Be-EnM1_rev	GGAATGGTGGCAAAGGGCAGCGAAGTACCAATGAC	EnM$_1$-BeM$_2$-BaTC$_3$
Be-BassM1_rev	GGAATGGTGGCAAAGGGCATTGAGCTGCCACCAATAG	BaSYN-BeM$_2$
Ba-BeauM2_for	CTATTGGTGGCAGCTCAATGCCCTTTGCCACCATTCC	BaSYN-BeM$_2$
Be-BaC3ctd_for	CAATACATGGACCACACGCGCGGACCTGGTTGCGACTTTTGG	BeSYN-BaC$_{3CTD}$
Ba-BeC3ntd_rev	CCAAAAGTCGCAACCAGGTCCGCGCGTGTGGTCCATGTATTG	BeSYN-BaC$_{3CTD}$
Ba-BeC3ctd_for	GTACGTGGACCATACTCGAAAAGCCGGCTGTGACTTTTGG	BeSYN-BaC$_{3NTD}$
Be-BaC3ntd_rev	CCAAAAGTCACAGCCGGCTTTTCGAGTATGGTCCACGTACTG	BeSYN-BaC$_{3NTD}$
Be-EnM2_for	CGCCCTCGACCATCACGACGCCATACAGCCTTATTC	BeM$_1$-EnM$_2$-BaTC$_3$
En-BeauM1_rev	GAATAAGGCTGTATGGCGTCGTGATGGTCGAG	BeM$_1$-EnM$_2$-BaTC$_3$
A. niger		
pVG-EnM1_for	GCAGACATCACCGTTTACCATGTCACTCCACACCCCAAG	EnSYN-BaTC$_3$
pVG-BeauM1_for	GAGCAGACATCACCGTTTACCATGGAGCCGCTCAAAAATG	BeSYN-BaTC$_3$
pVG-BassC3_rev	GGTCGGCATCTACTGTTTTCATAAAGACGCATTCAAAG	EnSYN-BaTC$_3$<br>BeSYN-BaTC$_3$
pVG-BassM1_for	GAGCAGACATCACCGTTTACCATGGAGCCACCCAACAACGC	BaSYN-EnTC$_3$<br>BaSYN-BeTC$_3$
pVG-EnC3_rev	CGGTCGGCATCTACTGTTTCTACAAAGCCTCGTTCAAAC	BaSYN-EnTC$_3$
pVG-BeauC3_rev	CGGTCGGCATCTACTGTTTTCACAAAGCCGAGTTTAGAC	BaSYN-BeTC$_3$
pVG2.2_for	AAACAGTAGATGCCGACCGGGATCC	pVG2.2 backbone
pVG2.2_rev	GGTAAACGGTGATGTCTGCTCAAG	pVG2.2 backbone

Supplementary Table 8: Primers used for clone verification.

Primer	Sequence 5' → 3'	Usage
EnSYNA2_for	CCGGTCTTGCGAGACTATCGTTGCCTATG	EnSYNA$_2$-fragment (652 bp)
EnSYNA2_rev	CTCCACGGCTTCCTTGGTATCGAACCTCTC	EnSYNA$_2$-fragment (652 bp)
BeSYNA2_for	AGCAAACCTGGCATATCTGCCGCTTGATCC	BeSYNA$_2$-fragment (685 bp)
BeSYNA2_rev	ATTCTCGGTCGGGCCGTAGGCGTTGTAG	BeSYNA$_2$-fragment (685 bp)
BaSYNA2_for	CTTCCTCGGCATCCTCAAAGCAAATCTGG	BaSYNA$_2$-fragment (640 bp)
BaSYNA2_rev	AGGGTCGAATCTATCGCCACCAGAGATG	BaSYNA$_2$-fragment (640 bp)

Supplementary Table 9: Plasmids used and generated in this study.

Plasmid	Features	Reference
pGEX4T1	tac-expression vector conferring N-terminal GST-tag	GE Healthcare, Freiburg, Germany
pET28a	T7-expression vector conferring N-terminal His$_6$-tag	Novagen, Merck KGaA, Darmstadt, Germany
pACYC(DUET-1)_BeSYN	T7-expression vector conferring N-terminal His$_6$-tag, with *besyn* (EU886196) from *Beauveria bassiana* ATCC7159	Matthes et al. (2012)
pJET1.2_BaSYN	Cloning vector, with *basyn* (FJ439897) from *Beauveria bassiana* ATCC 7159	This study
pACYC(DUET-1)_npgA	T7-expression vector conferring N-terminal His$_6$-tag, with *npgA* from *Aspergillus nidulans* (DSM 3365)	This study
pACYC(DUET-1)_sfp	T7-expression vector conferring N-terminal His$_6$-tag, with *sfp* from *Bacillus subtilis ssp. spizizenii* (ATCC6633)	This study
pACYC(DUET-1)_svp	T7-expression vector conferring N-terminal His$_6$-tag, with *svp* from *Streptomyces mobaraensis* (DSM40903)	This study
pGEX4T1_EnSYN	pGEX4T1 with *ensyn* (KP000028) of *Fusarium oxysporum* ETH 1536	This study
pGEX4T1_BeSYN	pGEX4T1 with *besyn* (EU886196) from *Beauveria bassiana* ATCC7159	This study
pGEX4T1_BaSYN	pGEX4T1 with *basyn* (FJ439897) from *Beauveria bassiana* ATCC 7159	This study
pGEX4T1_EnSYN-BaTC$_3$	pGEX4T1 with hybrid synthetase EnSYN-BaTC$_3$	This study
pGEX4T1_BaSYN-EnTC$_3$	pGEX4T1 with hybrid synthetase BaSYN-EnTC$_3$	This study
pGEX4T1_BaSYN-BeTC$_3$	pGEX4T1 with hybrid synthetase BaSYN-BeTC$_3$	This study
pGEX4T1_BeSYN-BaTC$_3$	pGEX4T1 with hybrid synthetase BeSYN-BaTC$_3$	This study
pGEX4T1_EnSYN-BaC$_3$	pGEX4T1 with hybrid synthetase EnSYN-BaC$_3$	This study
pGEX4T1_BaSYN-EnC$_3$	pGEX4T1 with hybrid synthetase BaSYN-EnC$_3$	This study
pGEX4T1_BaSYN-BeC$_3$	pGEX4T1 with hybrid synthetase BaSYN-BeC$_3$	This study
pGEX4T1_BeSYN-BaC$_3$	pGEX4T1 with hybrid synthetase BeSYN-BaC$_3$	This study
pGEX4T1_EnSYN-BaC$_{3CTD}$	pGEX4T1 with hybrid synthetase EnSYN-BaC$_{3CTD}$	This study
pGEX4T1_EnSYN-BaC$_{3NTD}$	pGEX4T1 with hybrid synthetase EnSYN-BaC$_{3NTD}$	This study
pGEX4T1_EnSYN-BaC$_{3NTD+loop}$	pGEX4T1 with hybrid synthetase EnSYN-BaC$_{3NTD+bridging\ loop}$	This study
pGEX4T1_EnSYN-Ba$_{loop}$	pGEX4T1 with hybrid synthetase EnSYN-BaC$_{3bridging\ loop}$	This study
pGEX4T1_EnSYN-mutC$_3$	pGEX4T1 with hybrid synthetase EnSYN-mutC$_3$	This study

Plasmid	Features	Reference
pGEX4T1_EnSYNΔMt	pGEX4T1 with truncated synthetase EnSYNΔMt domain	This study
pGEX4T1_BaSYNΔMt	pGEX4T1 with truncated synthetase BaSYNΔMt domain	This study
pGEX4T1_EnSYNΔC_1	pGEX4T1 with truncated synthetase EnSYNΔC_1	This study
pGEX4T1_BeSYNΔC_1	pGEX4T1 with truncated synthetase BeSYNΔC_1	This study
pGEX4T1_BaSYNΔC_1	pGEX4T1 with truncated synthetase BaSYNΔC_1	This study
pGEX4T1_EnSYNΔC_3	pGEX4T1 with truncated synthetase EnSYNΔC_3	This study
pGEX4T1_EnSYNΔC_1C_3	pGEX4T1 with truncated synthetase EnSYNΔC_1C_3	This study
pET28a_EnSYN-C_3	pET28a with EnSYN-C_3 domain	This study
pET28a_BaSYN-C_3	pET28a with BaSYN-C_3 domain	This study
pGEX4T1_BaSYN-EnM_2	pGEX4T1 with hybrid synthetase BaSYN-EnM_2	This study
pGEX4T1_EnSYN-BaM_2	pGEX4T1 with hybrid synthetase EnSYN-BaM_2	This study
pGEX4T1_EnSYNΔ$Ppant_{2a}$	pGEX4T1 with synthetase variant EnSYN-S2538A	This study
pGEX4T1_EnSYNΔ$Ppant_{2b}$	pGEX4T1 with synthetase variant EnSYN-S2632A	This study
pGEX4T1_BeM_1-BaM_2-EnTC_3	pGEX4T1 with hybrid synthetase BeM_1-BaM_2-EnTC_3	This study
pGEX4T1_EnM_1-BeM_2-BaTC_3	pGEX4T1 with hybrid synthetase EnM_1-BeM_2-BaTC_3	This study
pGEX4T1_BaSYN-BeM_2	pGEX4T1 with hybrid synthetase BaSYN-BeM_2	This study
pGEX4T1_BeSYN-BaC_{3CTD}	pGEX4T1 with hybrid synthetase BeSYN-BaC_{3CTD}	This study
pGEX4T1_BeSYN-BaC_{3NTD}	pGEX4T1 with hybrid synthetase BeSYN-BaC_{3NTD}	This study
pGEX4T1_BeM_1-EnM_2-BaTC_3	pGEX4T1 with hybrid synthetase BeM_1-EnM_2-BaTC_3	This study
A. niger		
pVG2.2	PgpdA::rtTA::TcgrA-tetO7::Pmin::TtrpC-pyrG*	Meyer *et al.* (2011)
pDS4.2	pVG2.2 with *ensyn* (KP000028) from *Fusarium oxysporum* ETH 1536	Richter *et al.* (2014)
pVG2.2_EnSYN-BaTC_3	pVG2.2 with hybrid synthetase EnSYN-BaTC_3	This study
pVG2.2_BeSYN-BaTC_3	pVG2.2 with hybrid synthetase BeSYN-BaTC_3	This study
pVG2.2_BaSYN-EnTC_3	pVG2.2 with hybrid synthetase BaSYN-EnTC_3	This study
pVG2.2_BaSYN-BeTC_3	pVG2.2 with hybrid synthetase BaSYN-BeTC_3	This study

Supplementary Table 10: Strains used and generated in this study.

Strain	Features	Reference
E.coli		
E. coli BL21 gold (DE3)	*F- ompT hsdS(rB*$^{-}$ *mB*$^{-}$*) dcm*$^{+}$ *Tetr gal* λ(DE3) *endA Hte*	Stratagene (Agilent Technologies), Waldbronn, Germany
E. coli DH5α (K12)	Δ*araBAD*, Δ*rhaBAD*	Datsenko & Wanner (2000)
BL21_EnSYN-BaTC_3	*E. coli* BL21 gold (DE3) with pGEX4T1_EnSYN-BaTC_3	This study
BL21_sfp_EnSYN-BaTC_3	*E. coli* BL21 gold (DE3) with pACYC(DUET-1)_sfp and pGEX4T1_EnSYN-BaTC_3	This study
BL21_svp_EnSYN-BaTC_3	*E. coli* BL21 gold (DE3) with pACYC(DUET-1)_svp and pGEX4T1_EnSYN-BaTC_3	This study
BL21_npgA_EnSYN-BaTC_3	*E. coli* BL21 gold (DE3) with pACYC(DUET-1)_ngpA and pGEX4T1_EnSYN-BaTC_3	This study
BL21_npgA_BaSYN-EnTC_3	*E. coli* BL21 gold (DE3) with pACYC(DUET-1)_ngpA and pGEX4T1_BaSYN-EnTC_3	This study
BL21_npgA_BeSYN-BaTC_3	*E. coli* BL21 gold (DE3) with pACYC(DUET-1)_ngpA and pGEX4T1_BeSYN-BaTC_3	This study
BL21_npgA_BaSYN-BeTC_3	*E. coli* BL21 gold (DE3) with pACYC(DUET-1)_ngpA and pGEX4T1_BaSYN-BeTC_3	This study
BL21_npgA_EnSYN-BaC_3	*E. coli* BL21 gold (DE3) with pACYC(DUET-1)_ngpA and pGEX4T1_EnSYN-BaC_3	This study
BL21_npgA_BaSYN-EnC_3	*E. coli* BL21 gold (DE3) with pACYC(DUET-1)_ngpA and pGEX4T1_BaSYN-EnC_3	This study
BL21_npgA_BeSYN-BaC_3	*E. coli* BL21 gold (DE3) with pACYC(DUET-1)_ngpA and pGEX4T1_BeSYN-BaC_3	This study
BL21_npgA_BaSYN-BeC_3	*E. coli* BL21 gold (DE3) with pACYC(DUET-1)_ngpA and pGEX4T1_BaSYN-BeC_3	This study
BL21_npgA_EnSYN-BaC_{3CTD}	*E. coli* BL21 gold (DE3) with pACYC(DUET-1)_ngpA and pGEX4T1_EnSYN-BaC_{3CTD}	This study
BL21_npgA_EnSYN-BaC_{3NTD}	*E. coli* BL21 gold (DE3) with pACYC(DUET-1)_ngpA and pGEX4T1_EnSYN-BaC_{3NTD}	This study
BL21_npgA_EnSYN-Ba$C_{3NTD+loop}$	*E. coli* BL21 gold (DE3) with pACYC(DUET-1)_ngpA and pGEX4T1_EnSYN-Ba$C_{3NTD+bridging\ loop}$	This study
BL21_npgA_EnSYN-Ba_{loop}	*E. coli* BL21 gold (DE3) with pACYC(DUET-1)_ngpA and pGEX4T1_EnSYN-Ba$C_{3bridging\ loop}$	This study
BL21_npgA_EnSYN-mutC_3	*E. coli* BL21 gold (DE3) with pACYC(DUET-1)_ngpA and pGEX4T1_EnSYN-mutC_3	This study
BL21_npgA_EnSYNΔMt	*E. coli* BL21 gold (DE3) with pACYC(DUET-1)_ngpA and pGEX4T1_EnSYNΔMt	This study
BL21_npgA_EnSYN-BaM_2	*E. coli* BL21 gold (DE3) with pACYC(DUET-1)_ngpA and pGEX4T1_EnSYN-BaM_2	This study
BL21_npgA_BaSYN-EnM_2	*E. coli* BL21 gold (DE3) with pACYC(DUET-1)_ngpA and pGEX4T1_BaSYN-EnM_2	This study
BL21_npgA_BaSYNΔMt	*E. coli* BL21 gold (DE3) with pACYC(DUET-1)_ngpA and pGEX4T1_BaSYNΔMt	This study
BL21_npgA_EnSYNΔC_1	*E. coli* BL21 gold (DE3) with pACYC(DUET-1)_ngpA and pGEX4T1_EnSYNΔC_1	This study
BL21_npgA_BeSYNΔC_1	*E. coli* BL21 gold (DE3) with pACYC(DUET-1)_ngpA and pGEX4T1_BeSYNΔC_1	This study
BL21_npgA_BaSYNΔC_1	*E. coli* BL21 gold (DE3) with pACYC(DUET-1)_ngpA and pGEX4T1_BaSYNΔC_1	This study
BL21_npgA_EnSYNΔC_3	*E. coli* BL21 gold (DE3) with pACYC(DUET-1)_ngpA and pGEX4T1_EnSYNΔC_3	This study
BL21_npgA_EnSYNΔC_1C_3	*E. coli* BL21 gold (DE3) with pACYC(DUET-1)_ngpA and pGEX4T1_EnSYNΔC_1C_3	This study
BL21_npgA_EnSYNΔC_3+EnC_3	*E. coli* BL21 gold (DE3) with pACYC(DUET-1)_ngpA, pGEX4T1_EnSYNΔC_3 and pET28a_EnSYN-C_3	This study

Strain	Features	Reference
BL21_npgA_EnSYNΔC_3+BaC_3	*E. coli* BL21 gold (DE3) with pACYC(DUET-1)_ngpA, pGEX4T1_EnSYNΔC_3 and pET28a_BaSYN-C_3	This study
BL21_npgA_EnSYNΔC_1C_3+EnC_3	*E. coli* BL21 gold (DE3) with pACYC(DUET-1)_ngpA, pGEX4T1_EnSYNΔC_1C_3 and pET28a_EnSYN-C_3	This study
BL21_npgA_EnSYNΔC_1C_3+BaC_3	*E. coli* BL21 gold (DE3) with pACYC(DUET-1)_ngpA, pGEX4T1_EnSYNΔC_1C_3 and pET28a_BaSYN-C_3	This study
BL21_npgA_ EnSYNΔ$Ppant_{2a}$	*E. coli* BL21 gold (DE3) with pACYC(DUET-1)_ngpA, pGEX4T1_ EnSYNΔ$Ppant_{2a}$	This study
BL21_npgA_ EnSYNΔ$Ppant_{2b}$	*E. coli* BL21 gold (DE3) with pACYC(DUET-1)_ngpA, pGEX4T1_ EnSYNΔ$Ppant_{2b}$	This study
BL21_npgA_BeM_1-BaM_2-EnTC_3	*E. coli* BL21 gold (DE3) with pACYC(DUET-1)_ngpA and pGEX4T1_BeM_1-BaM_2-EnTC_3	This study
BL21_npgA_EnM_1-BeM_2-BaTC_3	*E. coli* BL21 gold (DE3) with pACYC(DUET-1)_ngpA and pGEX4T1_EnM_1-BeM_2-BaTC_3	This study
BL21_npgA_BaSYN-BeM_2	*E. coli* BL21 gold (DE3) with pACYC(DUET-1)_ngpA and pGEX4T1_BaSYN-BeM_2	This study
BL21_npgA_BeSYN-BaC_{3CTD}	*E. coli* BL21 gold (DE3) with pACYC(DUET-1)_ngpA and pGEX4T1_BeSYN-BaC_{3CTD}	This study
BL21_npgA_BeSYN-BaC_{3NTD}	*E. coli* BL21 gold (DE3) with pACYC(DUET-1)_ngpA and pGEX4T1_BeSYN-BaC_{3NTD}	This study
BL21_npgA_BeM_1-EnM_2-BaTC_3	*E. coli* BL21 gold (DE3) with pACYC(DUET-1)_ngpA and pGEX4T1_BeM_1-EnM_2-BaTC_3	This study
A. niger		
AB 1.13	*pyrG*$^-$, *prtT*$^-$	Mattern *et al.* (1992)
MA 169.4	*pyrG*$^-$, *kusA*$^-$	Carvalho *et al.* (2010)
CS2.6	AB1.13 with pVG2.2_BeSYN-BaTC_3	This study
CS5.3	MA 169.4 with pVG2.2_EnSYN-BaTC_3	This study
CS6.15	MA 169.4 with pVG2.2_BaSYN-EnTC_3	This study
CS7.1/3/8	MA 169.4 with pVG2.2_BaSYN-BeTC_3	This study
**Bioactivity strains**		
Trypanosoma brucei rhodesiense	STIB 900 (trypomastigotes)	Swiss TPH
Trypanosoma cruzi	Tulahuen C4 (amastigotes)	Swiss TPH
Leishmania donovani	MHOM-ET-67/L82 (host free axenic amastigotes)	Swiss TPH
Bacillus subtilis	JMRC-No. STI:10880	Jena Microbial Resource Collection (JMRC)
Pseudomonas aeruginosa	JMRC-No. ST:337721	JMRC
Staphylococcus aureus	JMRC-No. ST:33793 (multi-resistent)	JMRC
Enterococcus faecalis	JMRC-No. ST:33700 (vancomycin-resistent)	JMRC
Mycobacterium vaccae	JMRC-No. STI:10670	JMRC
Sporobolomyces salmonicolor	JMRC-No. ST:35974	JMRC
Candida albicans	JMRC-No. STI:25000	JMRC
Penicillium notatum	JMRC-No. STI:50164	JMRC
Aspergillus fumigatus	JMRC-No. Afum:00073 = ATCC46645	JMRC
**Cell lines**		
L6	Rat-derived L6 cell line	Swiss TPH

Supplementary Table 11: Expected band sizes for colony PCR of wild-type, truncated and hybrid synthetases.

**EnSYNA$_2$-fragment (652 bp)**	**BaSYNA$_2$-fragment (640 bp)**	**BeSYNA$_2$-fragment (685 bp)**	**EnSYNC$_3$ (1414 bp)**	**BaSYNC$_3$ (1411 bp)**
EnSYN	BaSYN	BeSYN	EnSYN-C$_3$	BaSYN-C$_3$
EnSYN-BaC$_{3NTD}$	BaSYN-BeTC$_3$	BeSYN-BaTC$_3$		
EnSYNΔC$_1$C$_3$	BeM$_1$-BaM$_2$-EnTC$_3$	EnM$_1$-BeM$_2$-BaTC$_3$		
EnSYN-BaC$_3$	BaSYN-EnC$_3$	BeSYNΔC$_1$		
EnSYN-BaC$_{3NTD+loop}$	BaSYN-BeC$_3$	BeSYN-BaC$_3$		
EnSYN-Ba$_{loop}$	EnSYN-BaM$_2$	BaSYN-BeM$_2$		
EnSYNΔC$_1$	BaSYNΔMt	BeSYN-BaC$_{3CTD}$		
EnSYNΔC$_3$	BaSYN-EnTC$_3$	BeSYN-BaC$_{3NTD}$		
EnSYN-BaTC$_3$	BaSYNΔC$_1$			
EnSYN-mutC$_3$				
EnSYNΔMt				
BaSYN-EnM$_2$				
EnSYN-BaC$_{3CTD}$				
EnSYNΔPpant$_{2a}$				
EnSYNΔPpant$_{2b}$				
BeM$_1$-EnM$_2$-BaTC$_3$				

Supplementary Table 12: Expected band sizes for control restriction of wild-type, truncated and hybrid synthetases.

**pGEX4T1_EnSYN**	**pGEX4T1_BeSYN**	**pGEX4T1_BaSYN**	**pGEX4T1_BaSYN-BeTC$_3$**	**pGEX4T1_BaSYN-EnTC$_3$**	**pGEX4T1_EnM$_1$-BeM$_2$-BaTC$_3$**	**pGEX4T1_BeM$_1$-BaM$_2$-EnTC$_3$**	**pGEX4T1_BaSYN-EnM$_2$**
PstI, SmaI	EcoRI, XhoI	PstI, BglII	PstI, SmaI	PstI, SmaI	XhoI	PstI, SmaI	BamHI, XhoI
7740	6930	4054	5325	5325	7726	5521	7729
2541	3061	2524	2618	2971	3940	2971	2416
1658	1932	2439	2439	2439	1314	2417	1730
1377	1105	1777	1559	1658	1035	1658	1230
1020	1083	1271	1447	1447	396	1447	651
	396	935	464	290		290	337
		395/4	290	143		143	270
		353	143	93			
		143	93				
		93					

**pGEX4T1_BeSYN-BaTC$_3$**	**pGEX4T1_EnSYN-BaC$_3$**	**pGEX4T1_BaSYN-EnC$_3$**	**pGEX4T1_BaSYN-BeC$_3$**	**pGEX4T1_BeSYN-BaC$_3$**	**pGEX4T1_EnSYNΔC$_1$C$_3$**	**pGEX4T1_BaSYNΔMt**	**pET28a_BaSYN-C$_3$**
AflII, BamHI, BglII	XhoI	PstI	SmaI, XhoI	BglII, XhoI	XhoI	SmaI, BglII	SmaI, BglII
7371	7726	5325	7529	7291	5862	5831	3895
3221	2901	3812	3157	3851	2901	4236	2046
2252	1526	2439	2258	1932	1526	1562	724
1125	1035	1658	1078	948	1035	935	
538	872	914	255	396	270	449	
	270	143	82	80		63	
			32				

**pGEX4T1_EnSYN-BaC$_{3NTD}$**	**pGEX4T1_EnSYN-BaC$_{3NTD+loop}$**	**pGEX4T1_EnSYN-Ba$_{loop}$**	**pGEX4T1_EnSYN-mutC$_3$**	**pGEX4T1_EnSYNΔMt**	**pGEX4T1_EnSYNΔPpant$_{2a}$**	**pGEX4T1_EnSYN-BaC$_{3CTD}$**	**pET28a_EnSYN-C$_3$**
XhoI	XhoI	XhoI	XhoI	XhoI	HindIII	XhoI	BglII, PstI, SmaI
7726	7726	8604	8604	8604	9471	8604	3895
2901	2901	2901	2901	2901	2536	2901	1927
1526	1526	1526	1526	1035	2329	1526	849
1035	1035	1035	1035	479		1035	
872	872	270	270			270	
270	270						

**pGEX4T1_EnSYN-BaTC$_3$**	**pGEX4T1_EnSYNΔC$_3$**	**pGEX4T1_EnSYNΔC$_1$**	**pGEX4T1_BeSYNΔC$_1$**	**pGEX4T1_BaSYNΔC$_1$**	**pGEX4T1_EnSYN-BaM$_2$**	**pGEX4T1_BeSYN-BaC$_{3NTD}$**	pGEX4T1_BaSYN-BeM$_2$
SmaI, XhoI	XhoI	PstI, SmaI	BglII, XhoI	XhoI, SmaI	XhoI	XhoI, BglII	XhoI, BglII
7726	7230	6372	5683	6361	8604	7087	5751
2901	2901	2541	4144	2944	3132	3851	3860
2370	1526	1658	1932	2258	1293	1932	2361
1035	1035	1377	948	1078	1035	948	1978
270	270	1020	396	255	255	396	396
					32	293	80

**pGEX4T1_EnSYNΔPpant$_{2b}$**	**pGEX4T1_BeSYN-BaC$_{3CTD}$**	**pGEX4T1_BeM$_1$-EnM$_2$-BaTC$_3$**
HindIII	XhoI, BglII	XhoI
9471	11222	8239
2536	1932	3519
2329	948	2416
	396	270

Supplementary Table 13: Gradient used for LC-ESI-MS measurements. Analysis was performed on an exactive mass spectrometer (Thermo Fisher Scientific, Dreieich, Germany). Column: Poroshell 120 EC-C18 2.7 µm (Agilent Technologies, Waldbronn, Germany). Flow rate: 0.3 mL/min. Solvent A: water (0.1 % HCOOH), Solvent B: acetonitrile (0.1 % HCOOH).

Time [min]	ACN [%]
0	50
10	100
13	100
13.01	50
15	50

Supplementary Table 14: Gradient used for LC-ESI-IDA measurements. Analysis was performed on an Orbitrap XL mass spectrometer (Thermo Fisher, Dreieich, Germany), Column: Vydac 218MS C18 5u 150mm ID2.1 mm (GRACE, Worms, Germany). Flow rate: 0.3 mL/min. Solvent A: water (0.1 % HCOOH), Solvent B: acetonitrile (0.1 % HCOOH), IDA mode (TOP3).

Time [min]	ACN [%]
0	5
1	5
20	45
30	99
35	99
35.1	5
39	5

Supplementary Table 15: Gradient used for LC-ESI-MS/MS and -MRM measurements. Analysis was performed on an ESI-Triple-Quadrupole mass spectrometer (6460 Series, Agilent Technologies, Waldbronn, Germany; UHPLC 1290 Infinity-Series (Agilent Technologies, Waldbronn, Germany); Column: Poroshell 120 EC-C18 3.0x50 mm (Agilent Technologies, Waldbronn, Germany); Flow rate: 0.4 mL/min. Solvent A: water, Solvent B: isopropanol.

Time [min]	ACN [%]
0.0	50
2.5	80
2.6	100
3.6	100
3.9	5
4.9	5
5.0	50
6.0	50

Supplementary Table 16: Parameters used for LC-ESI-MS/MS and -MRM measurements. Analysis was performed on an ESI-Triple-Quadrupole mass spectrometer (Agilent Technologies, Waldbronn, Germany).

Parameter	**Bassianolide**	**Octa-enniatin B**	**Desmethyl-enniatin B**	**Desmethyl-bassianolide**	**Octa-beauvericin**	**Hexa-bassianolide**
Nozzle voltage [V]	1300	1500	1500	1500	1600	1500
Capillary [V]	3500	3000	3000	3000	3000	3000
Fragmentor	135	270	270	270	330	270
Parent ion [*m/z*]	931.0	875.5	620.2	875.5	1067.5	704.5
Collision energy	75	70	55	65	73	70
Daughter ions (MRM) [*m/z*]	350.1	549.0<br>336.2	-	-	645.3<br>384.2	577.2<br>350.1

Supplementary Table 17: Gradients used for CDP purification by flash chromatography. Reveleris Flash System (GRACE, Worms, Germany). Column: Reveleris C18-WP flash cartridge, 40 g (GRACE, Worms, Germany). Flow rate: 40 mL/min. Solvent A: water (0.1 % HCOOH), Solvent B: acetonitrile (0.1 % HCOOH).

**Octa-enniatin B**		**Octa-beauvericin**		**Hexa-bassianolide**	
Time [min]	ACN [%]	Time [min]	ACN [%]	Time [min]	ACN [%]
0	80	0	70	0	70
15	100	15	100	15	100
18	100	21	100	21	100

Supplementary Table 18: Gradients used for CDP purification by preparative HPLC. HPLC 1100 Series (Agilent Technologies, Waldbronn, Germany); Column: Grom-Sil 120 ODS-4 HE, 10 µm, 250 mm, ID:20 mm (GRACE, Worms, Germany); Flow rate: 15 mL/min; Solvent A: water (0.1 % HCOOH), Solvent B: acetonitrile (0.1 % HCOOH).

**Octa-enniatin B**		**Octa-beauvericin**		**Hexa-bassianolide**	
Time [min]	ACN [%]	Time [min]	ACN [%]	Time [min]	ACN [%]
0	80	0	80	0	70
20	100	5	80	1	90
25	100	20	100	19	100
25.01	80	25	100	22	100
27	80	25.01	80	22.1	70
		27	80	25	70

Supplementary Table 19: Gradient used for octa-enniatin B purification by analytical HPLC. HPLC 1200 series (Agilent Technologies, Waldbronn, Germany); Column: Luna 5u C18(2), 100 A, 100x4.6 mm, 5 micron (Phenomenex, City, Country); Flow rate: 1.5 mL/min; Solvent A: water (0.1 % HCOOH), Solvent B: acetonitrile (0.1 % HCOOH).

**Time [min]**	**ACN [%]**
0	50
10	100
13	100
13.01	50
15	50

Supplementary Table 20: Confirmation of expressed synthetases by tryptic digestion and peptide fingerprinting. Digested protein bands were measured on an Orbitrap XL mass spectrometer (Thermo Fisher, Dreieich, Germany). IDA-data were compared to in silico peptides using PeptideShaker.

Synthetase	Coverage [%]	Coverage Plot
General model		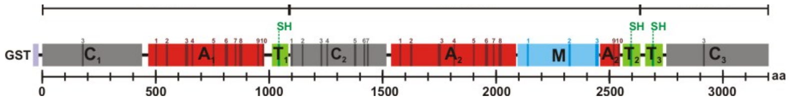
**Wild-type**		
EnSYN	27.1	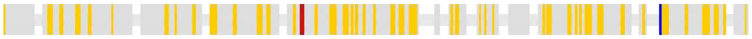
BeSYN	20.2	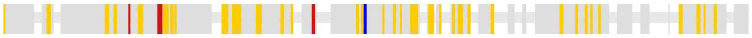
BaSYN	19.6	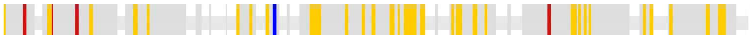
**Hybrid**		
EnSYN-BaTC$_3$	41.8	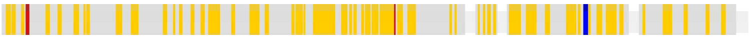
EnSYN-BaC$_3$	28.7	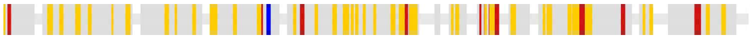
BaSYN-EnTC$_3$	22.5	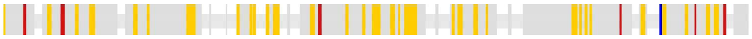
BaSYN-BeTC$_3$	32.1	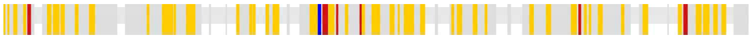
BaSYN-EnC$_3$	28.1	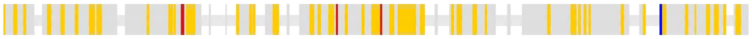
BeSYN-BaTC$_3$	34.7	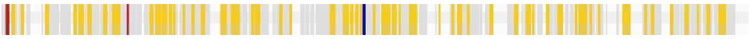
BeSYN-BaC$_3$	36.2	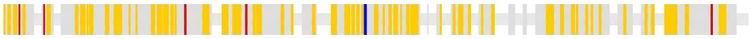
BaSYN-BeC$_3$	34.3	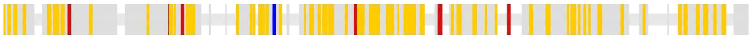
EnSYN-BaC$_{3NTD}$	14.0	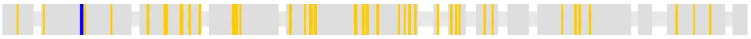
EnSYN-BaC$_{3CTD}$	22.0	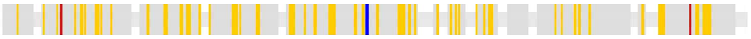
EnSYN-BaC$_{3loop}$	17.9	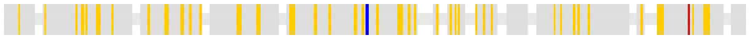
EnSYN-BaC$_{3NTD+loop}$	06.3	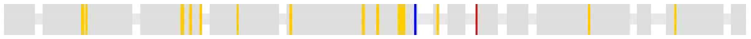
EnSYN-BaM$_2$	40.7	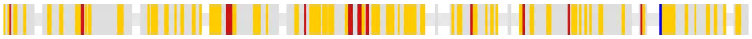
BaSYN-EnM$_2$	34.3	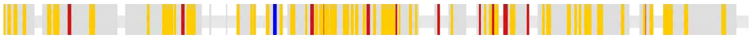
EnM$_1$-BeM$_2$-BaTC$_3$	13.5	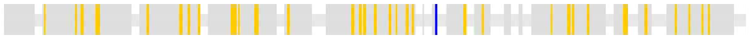
**Truncated**		
EnSYNΔC$_1$	25.0	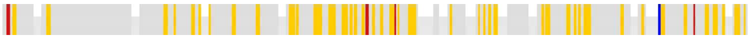
BeSYNΔC$_1$	37.4	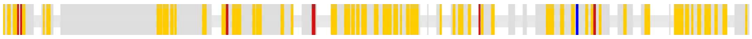
BaSYNΔC$_1$	19.5	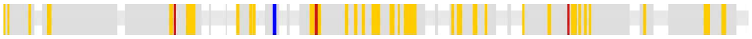
EnSYNΔMt	48.5	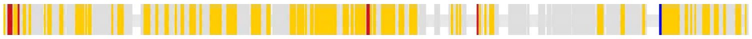
BaSYNΔMt	43.9	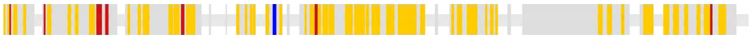

Supplementary Table 21: ^{1}H chemical shifts of hexa-bassianolide. Purified hexa-bassianolide was taken up in chloroform-d$_1$ and the spectrum was obtained on a Bruker 700 spectrometer (^{1}H: 700 MHz). The N-CH_3 signal at 3.10 ppm was used for integral calibration.

Position	δ_H [ppm], multiplicity (Integral; *J* [Hz])
**Leu**	
α	5.28, m (0.98)
β	1.73, m (1.01)
	1.65, m (1.03)
γ	1.45, m (1.03)
δ	0.94, d (2.97; 6.8)
	0.89, d (3.09; 6.8)
N-CH_3	3.10, s (3.00)
**D-Hiv**	
α	4.91, d (1.00; 8.38)
β	2.20, m (1.01)
γ	0.99, d (2.97; 6.64)
	0.92, d (2.84; 7.04)

Supplementary Table 22: ^{1}H and ^{13}C chemical shifts of octa-beauvericin. Despite the repetitive sequence, two resonance sets were observed due to the fact that octa-beauvericin adopts i) a distinct, asymmetrical conformation, in which each repeating unit gives unique chemical shifts (indices a, b, c, d), and ii) an ensemble of conformers that interconvert rapidly giving rise to degenerate chemical shifts (designated as 'ensemble average'; approx. 70 % abundance with respect to the distinct conformer). The signals of the Hα atom of D-Hiv$_d$ and the *N*-Me moieties were used for separate integral calibration of the asymmetrical conformer and the conformational ensemble, respectively.

Position		δ_C [ppm]	δ_H [ppm], multiplicity (Integral; *J* [Hz])
**Phe$_a$**			
	α	56.8	5.85, dd (2.29; 12.8, 4.4)[1]
	β	34.2	3.53, dd (4.81; 15.3, 4.4)[2]
		34.3	2.96, dd (2.97; 13.7, 13.5)[3]
	γ	137.1	-
	δ	126.8/126.9	7.20/7.26, m (30.60)[4]
	ε	128.6	7.23/7.26, m (30.60)[4]
	ζ	126.8/126.9	7.20/7.26, m (30.60)[4]
	C=O	169.8	-
	N-CH_3	31.8	3.24, s (2.79)
**D-Hiv$_a$**			
	α	74.4	5.30, d (0.94; 6.9)
	β	30.6	1.83, m (1.61)
	γ	17.9	0.76, d (2.96; 6.6)
		15.3	0.30, d (5.80; 6.8)[6]
	C'	170.2	-
**Phe$_b$**			
	α	60.1	4.81, dd (0.93; 11.4, 4.4)
	β	35.1	3.51, dd (4.81; 10.4, 4.3)[2]
		35.0	3.07, dd (1.03; 15.4, 11.4)
	γ	135.5	-
	δ	129.1	7.37, d (1.88; 7.7)[5]
		128.4	7.36, d (1.88; 7.6)[5]
	ε	128.6	7.23/7.26, m (30.60)[4]
	ζ	126.8/126.9	7.20/7.26, m (30.60)[4]
	C'	169.3	-
	N-CH_3	29.9	2.89 s (3.10)
**D-Hiv$_b$**			
	α	76.7	4.96, d (0.96; 2.6)
	β	28.3	1.61, m (2.86)[7]
	γ	18.8	0.72, d (3.35; 6.7)

Position		δ_C [ppm]	δ_H [ppm], multiplicity (Integral; J [Hz])
		17.6	0.36, d (2.85; 6.9)
	C‘	169.3	-
**Phe$_c$**			
	α	55.6	6.05, dd (1.95; 8.5, 4.5)
	β	35.1	2.82, dd (9.93; *)[12]
		35.7	3.61, dd (1.94; 14.5, 4.4)[8]
	γ	136.1	-
	δ	126.8/126.9	7.20/7.26, m (30.60)[4]
	ε	128.6	7.23/7.26, m (30.60)[4]
	ζ	126.8/126.9	7.20/7.26, m (30.60)[4]
	C‘	168.9	-
	N-CH_3	30.8	2.97 s (3.51)
**D-Hiv$_c$**			
	α	75.5	5.13, d (3.53; 6.6)[9]
	β	29.2	1.63, m (2.86)[7]
	γ	17.4	0.68, d (10.06; 6.7)[10]
		14.9	0.57, d (10.78; 6.7)[11]
	C‘	170.2	-
**Phe$_d$**			
	α	56.0	6.08, dd (1.95; 8.6, 4.7)
	β	35.0	3.64, dd (1.94; 14.7, 4.8)[8]
		35.8	2.84, dd (9.93; *)[12]
	γ	136.1	-
	δ	126.8/126.9	7.20/7.26, m (30.60)[4]
	ε	128.6	7.23/7.26, m (30.60)[4]
	ζ	126.8/126.9	7.20/7.26, m (30.60)[4]
	C‘	169.4	-
	N-CH_3	30.8	3.01, s (2.94)
**D-Hiv$_d$**			
	α	74.5	5.35, d (1.0; 1.9)
	β	29.3	1.32, m (3.75)
	γ	20.1	0.84, d (3.21; 6.9)
		15.3	0.30, d (5.8; 6.7)[6]
	C’	169.8	-

Position	δ_C [ppm]	δ_H [ppm], multiplicity (Integral; J [Hz])
**Phe$_{ensemble\ average}$**		
α	56.8	5.85, br s (4.78)[1]
β	34.2	3.53, dd (2.55; 15.3, 4.4)[2]
	34.3	2.96, dd (4.45; 13.7, 13.5)[3]
γ	136.8	-
δ	126.8/126.9	7.20/7.26, m (30.60)[4]
ε	128.6	7.23/7.26, m (30.60)[4]
ζ	126.8/126.9	7.20/7.26, m (30.60)[4]
C‘	169.9	-
N-CH_3	31.3	2.84, br s (12.00)[12]
**D-Hiv$_{ensemble\ average}$**		
α	75.4	5.12, br s (5.09)[9]
β	29.6	1.91, br s (4.58)
γ	17.6	0.70, br s (8.49)[10]
	18.1	0.57, br s (16.42)[11]
C’	168.9	-

* Coupling constants could not be determined due to signal overlap

[1-12] Signal overlap of the respective species

Supplementary Table 23: ^{1}H and ^{13}C chemical shifts of octa-enniatin B. Similarly to octa-beauvericin, the compound also gives rise to two resonance sets (see explanation Supplementary Table 22). However, for octa-enniatin B, the conformational ensemble is the main species (five times more abundant than the distinct conformer) as there is one strong ^{1}H-signal of the *N*-Me-group at 3.02 ppm and four minor signals. Owing to intense signal overlap, the N-CH_3 signal at 3.02 ppm was used for integral calibration of both species.

Position	δ_C [ppm]	δ_H [ppm], multiplicity (Integral; *J* [Hz])
**Val**		
α	60.3	5.51, d (0.41*)[1]
	60.9	5.50, d (0.41*)[1]
	62.5	4.91, br s (2.93)
	65.9	3.50, m (0.44)
β	27.5	2.35, m (4.74)
γ	18.8	1.02, d (44.27; 7.1)[2]
	18.1	1.02, d (44.27; 7.1)[2]
N-CH_3	32.2	3.21, s (0.63)
	33.7	3.12, s (1.20)[3]
	33.8	3.11, s (1.20)[3]
	32.3	3.02, s (12.0)
	30.0	2.97, s (0.63)
**D-Hiv**		
α	74.4	5.53, d (0.41; 5.9)[1]
	74.6	5.48, d (0.41; 7.8)[1]
	74.8	5.41, br s (0.10)
	79.9	5.02, d (4.29; 7.2)
β	29.7	2.24, m (4.83)
γ	20.1	1.04, d (44.27; 7.2)[2]
	19.0	0.89, d (13.90; 6.8)

* Coupling constants could not be defined due to signal overlaps
[1-3] Signal overlap of the respective species

3. Supplementary Figures

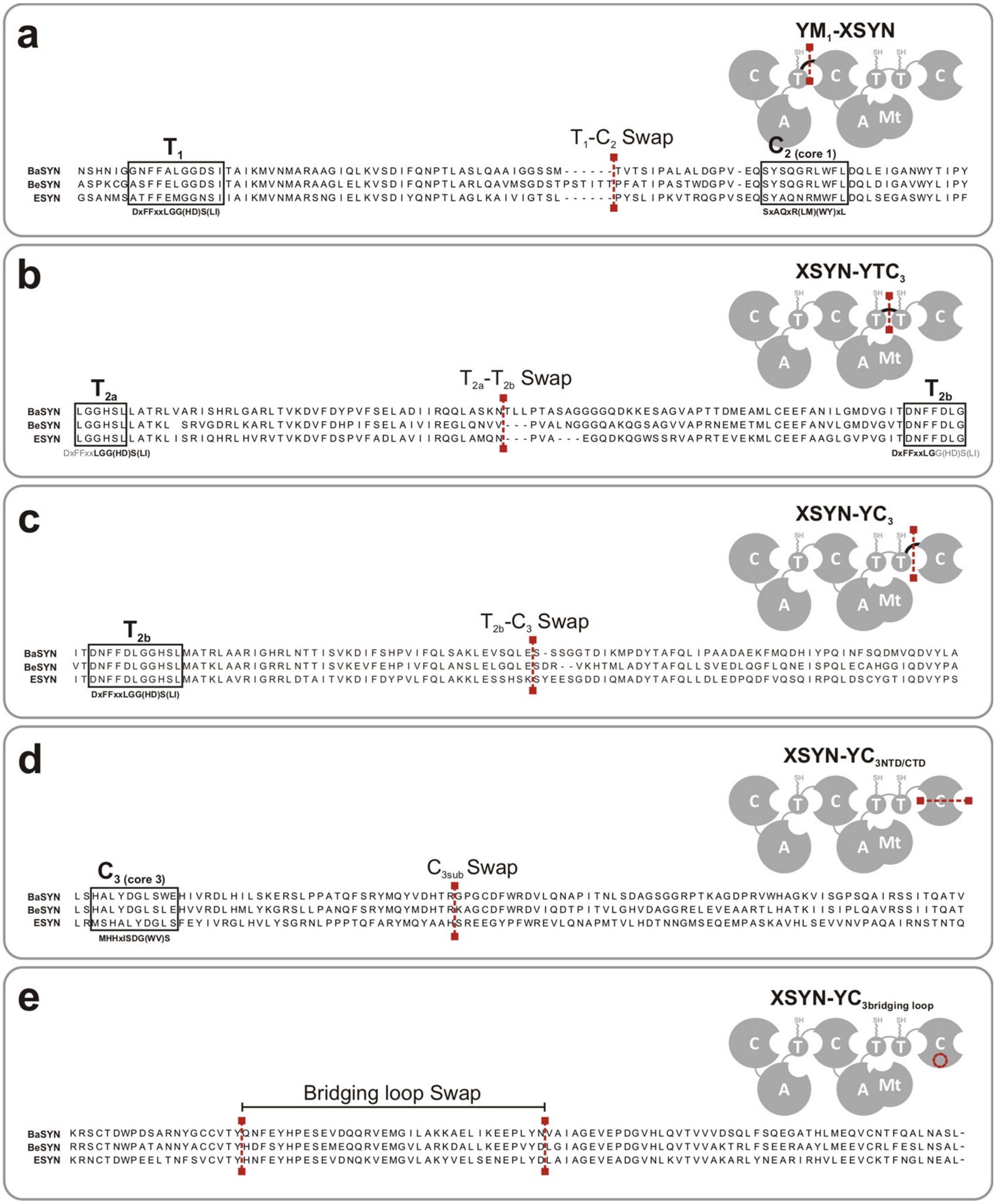

Supplementary Fig. 1: Synthetase swapping sites. (a) Swapping site (dotted line) in T_1-C_2-linker, (b) Swapping site in T_{2a}-T_{2b}-linker, (c) Swapping site in T_{2b}-C_3-linker, (d) Swapping site between C_{3NTD} and C_{3CTD}, (e) Swapping sites of C_3 bridging loop.

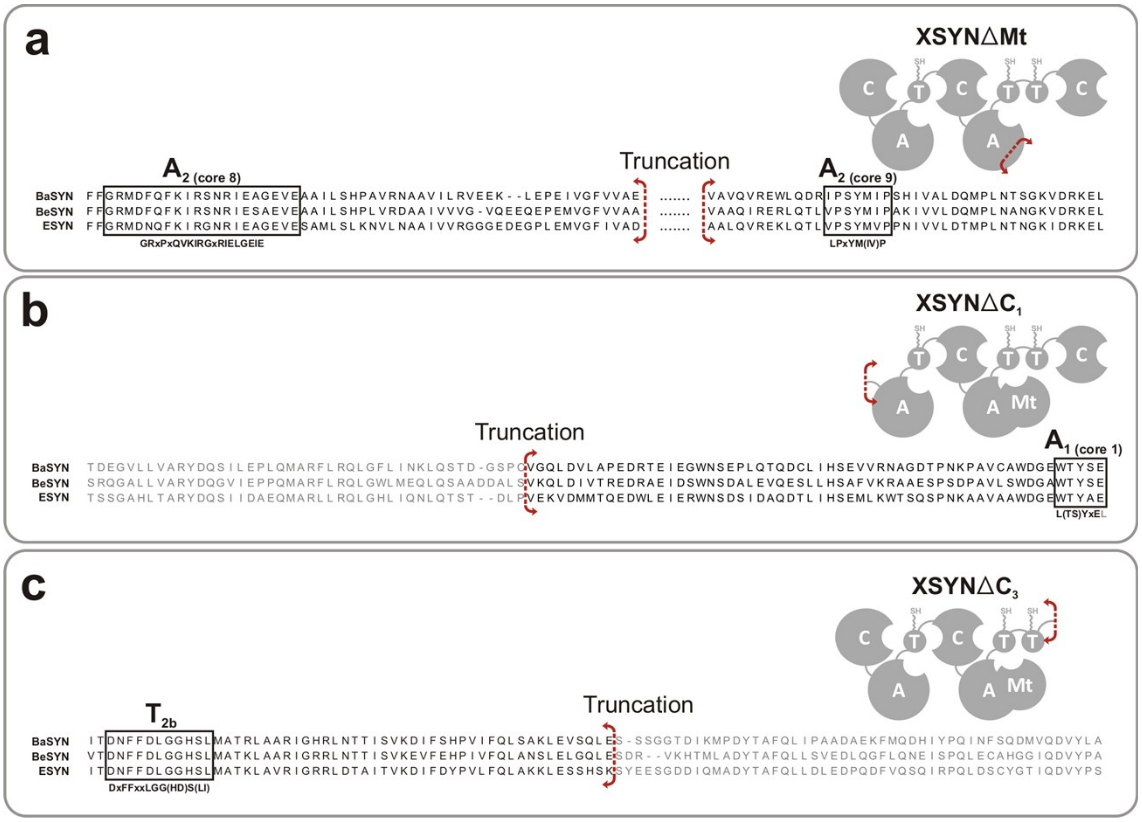

Supplementary Fig. 2: Synthetase truncation sites. (a) Truncation sites in XSYNΔMt, (b) Truncation site in XSYNΔC_1, (c) Truncation site in XSYNΔC_3.

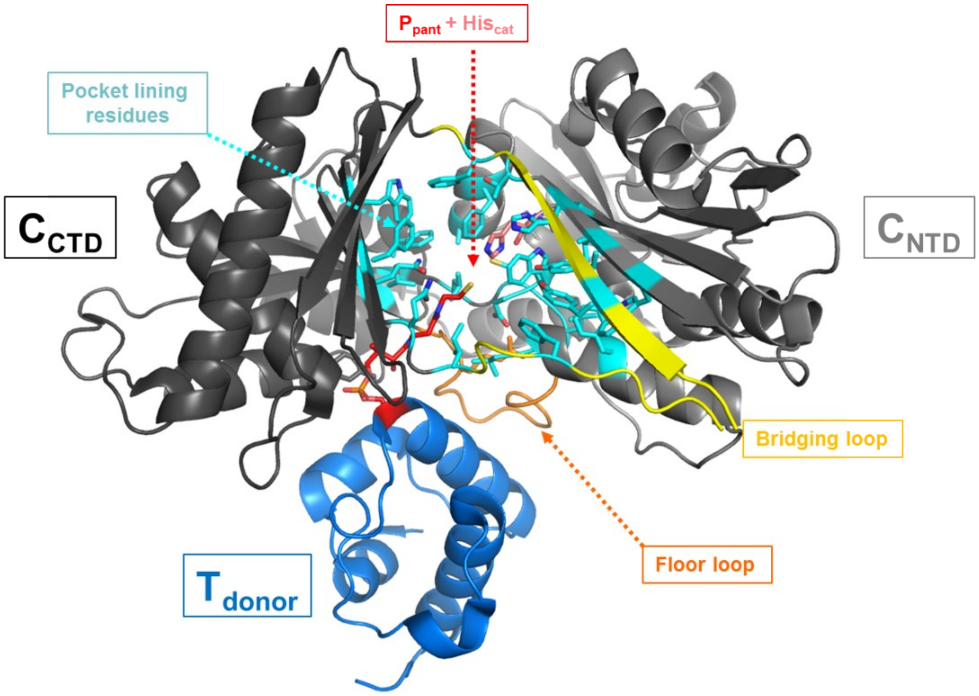

Supplementary Fig. 3: Crystal structure of the TqaA T-C_{term}-bidomain (PDB: 5EJD; Zhang *et al.* 2016). T domain (blue), C-terminal subdomain C_{CTD} (dark grey), N-terminal subdomain C_{NTD} (light grey), floor loop (orange), bridging loop (yellow), pocket lining residues (cyan), Ppant arm (red), catalytic His (light pink).

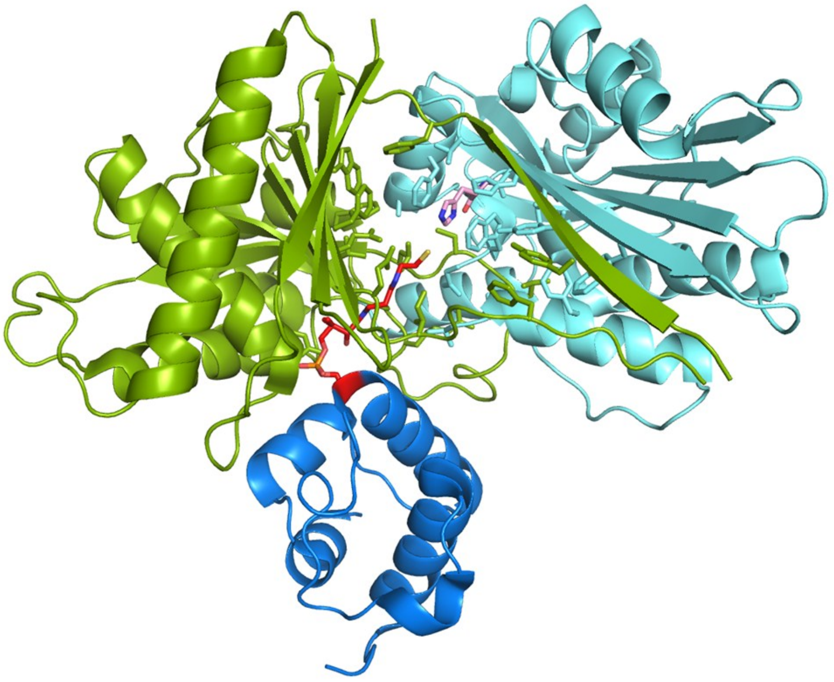

Supplementary Fig. 4: Illustration of the C_{CTD}-swap employing the TqaA T-C_{term} structure (Zhang *et al.* 2016). C_{CTD} (green) with floor and bridging loop crossing over to C_{NTD} (light blue), T_{don} domain (dark blue). Ppant arm and catalytic His (light pink).

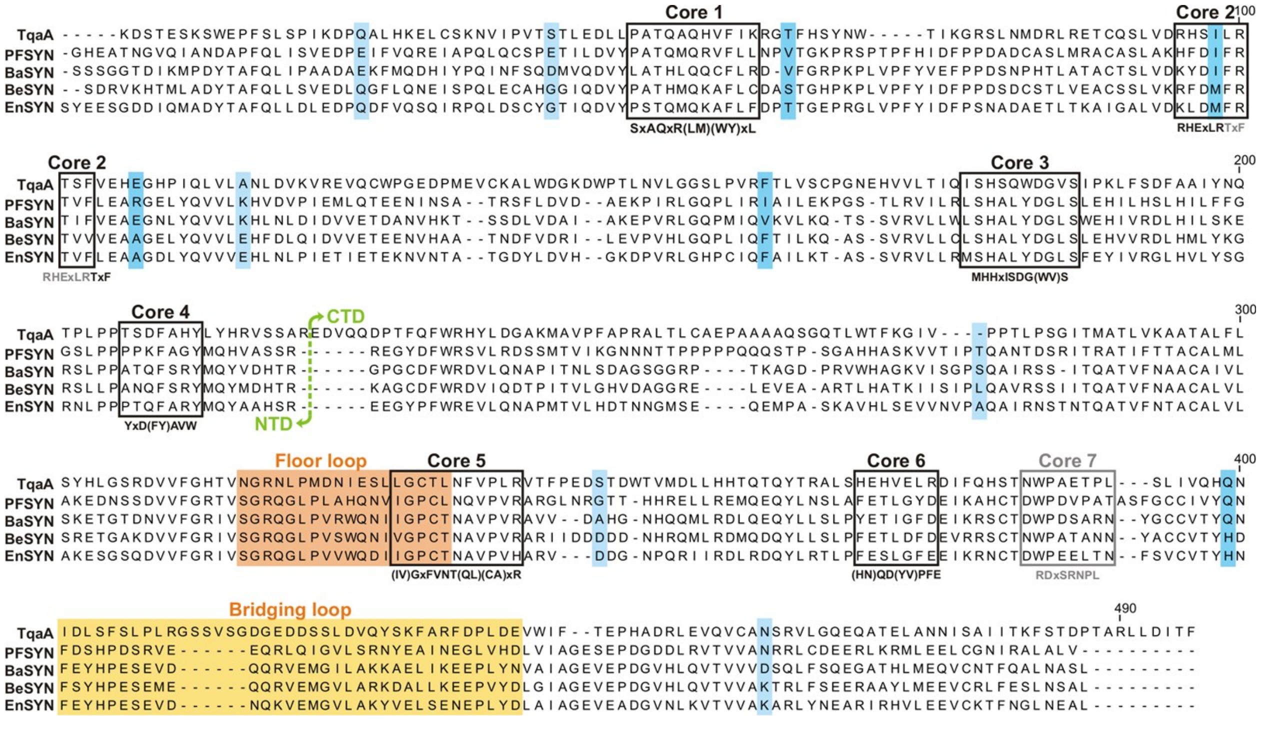

Supplementary Fig. 5: Alignment of C_3 sequences and TqaA-C_{term} sequence (Zhang *et al.* 2016). C domain core motifs (black frames), floor loop (orange), bridging loop (yellow), subdomain transition (green), 10 residues conserved for synthetases generating the same ring size (light blue + blue), swapped residues from BaSYN into EnSYN for generation of EnSYN-mutC_3 (blue).

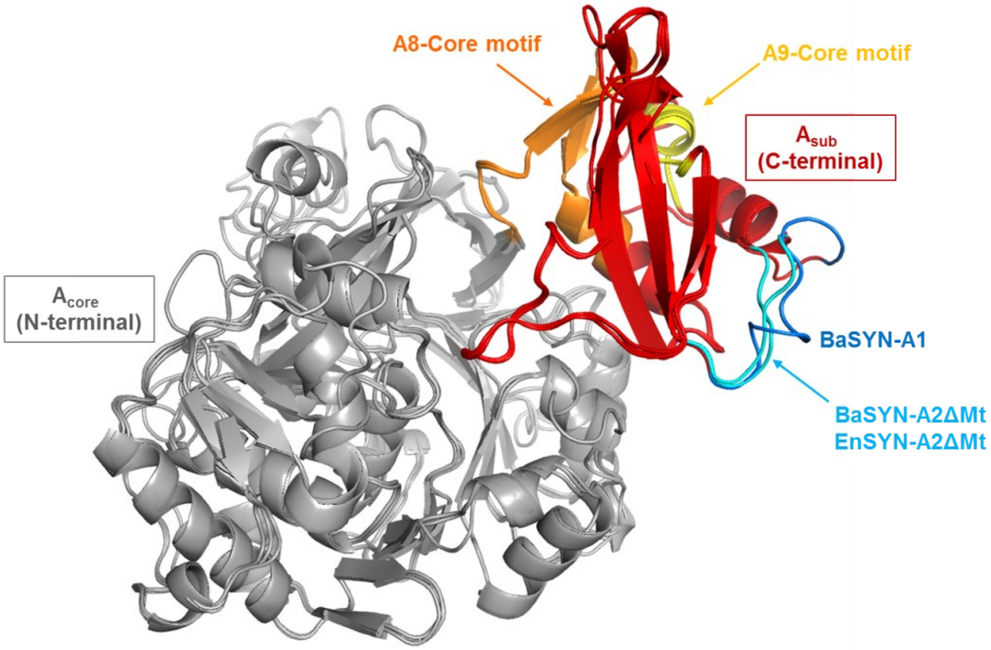

Supplementary Fig. 6: Superposition of structural models of A_1 and truncated $A_2\Delta Mt$ domains. Structure models were created with SWISS-MODEL on the basis of GrsA (PDB: 1AMU; Conti *et al.* 1997). N-terminal A_{core} subdomain (grey), C-terminal $\mathbf{A_{sub}}$ subdomain (red), A domain core motif 8 (orange), A domain core motif 9 (yellow), loop naturally containing the Mt domain insertion (light blue).

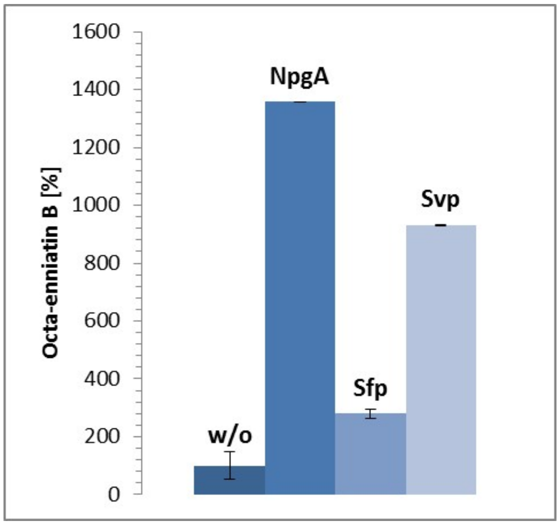

Supplementary Fig. 7: CDP production employing different PPTase genes. The relative yield of octa-enniatin B [%] by EnSYN-$BaTC_3$ was determined in *E. coli* (DE3) with the endogenous *E.coli* PPTase (w/o) in comparison to co-expression of the heterologous PPTases *npgA* (*Aspergillus nidulans*; DSM 3365), *sfp* (*Bacillus subtilis ssp. spizizenii*; ATCC6633) and *svp* (*Streptomyces mobaraensis*; DSM40903), respectively. LC-ESI-MRM-measurements were performed on an ESI-Triple-Quadrupole mass spectrometer.

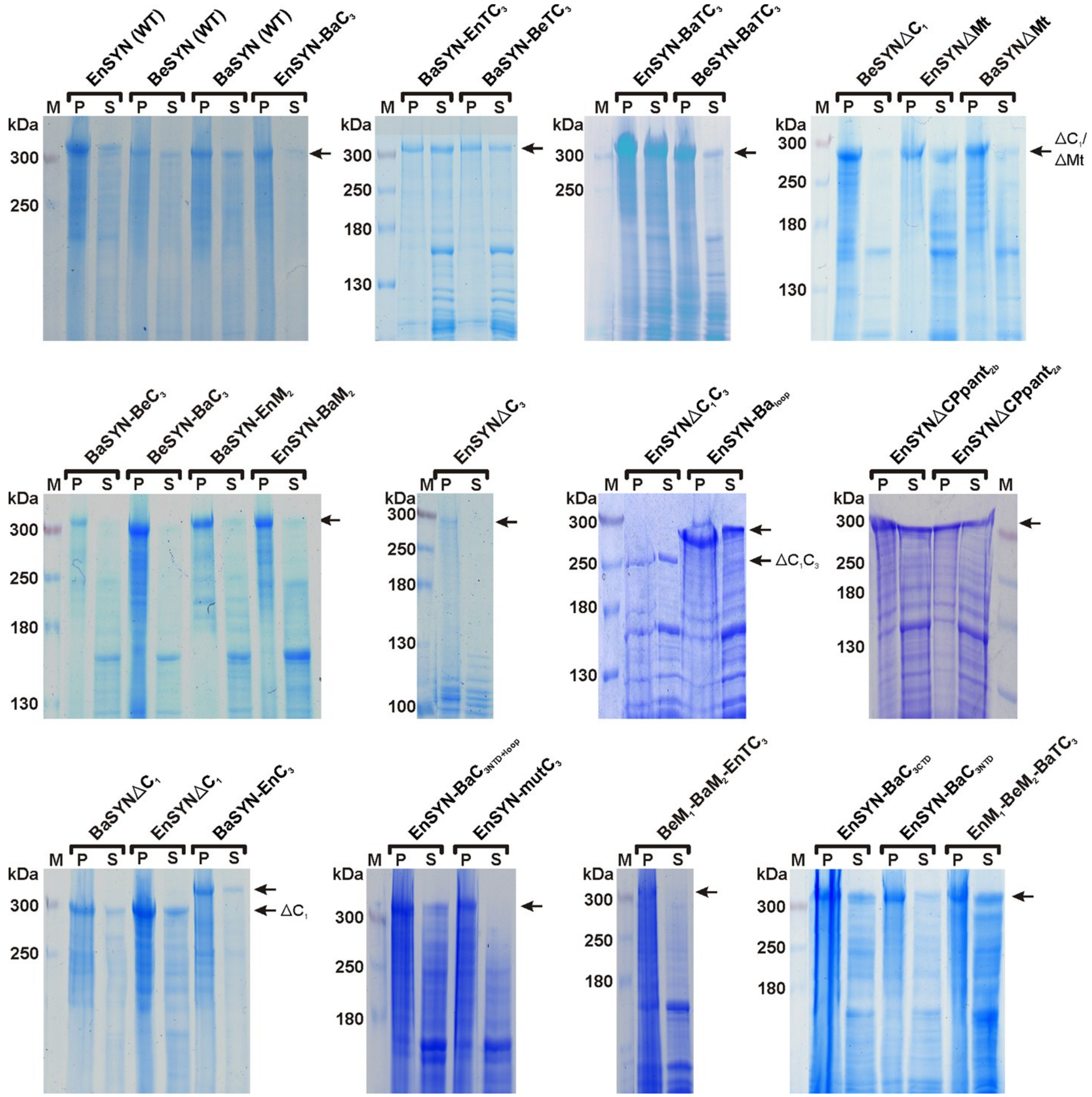

Supplementary Fig. 8: Expression of wild-type, truncated and hybrid synthetases in *E. coli* BL21 gold-NpgA. P: pellet (insoluble fraction), S: supernatant (soluble fraction). Arrow: Protein bands of GST-tagged synthetases.

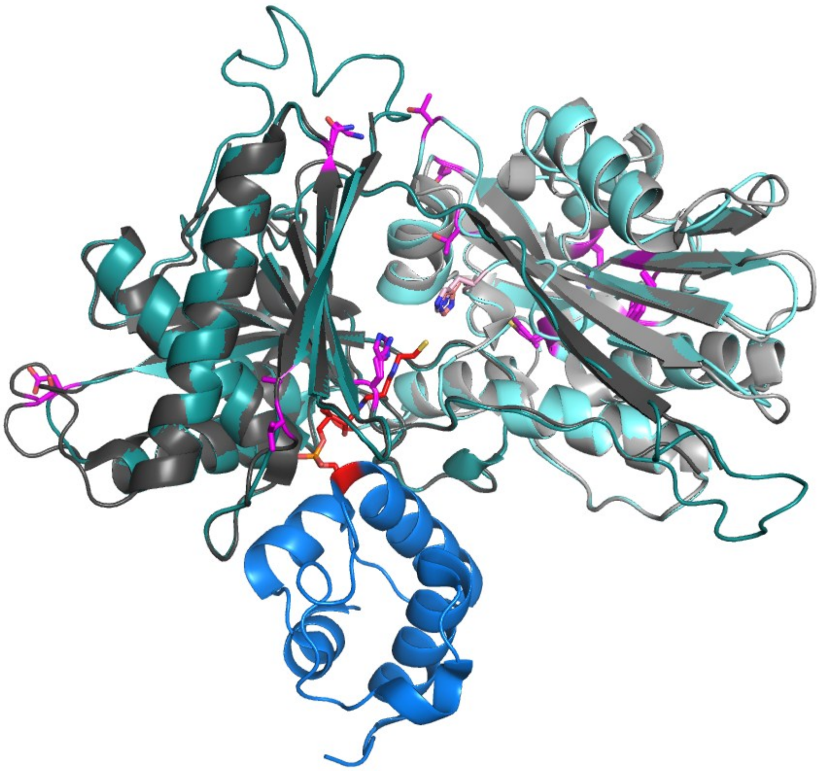

Supplementary Fig. 9: Superposition of TqaA T-C_{term} and a structural model of the EnSYN-C_3 domain. The model was created with SWISS-MODEL on the basis of TqaA (PDB: 5EJD; Zhang *et al.* 2016). T domain (blue), C-terminal subdomain C_{CTD} of TqaA (dark grey) and EnSYN-C_3 (dark cyan), N-terminal subdomain C_{NTD} of TqaA (light grey) and EnSYN-C_3 (cyan), Ppant arm (red), catalytic His (light pink), ten potential residues involved in ring size determination based on sequence alignments (magenta).

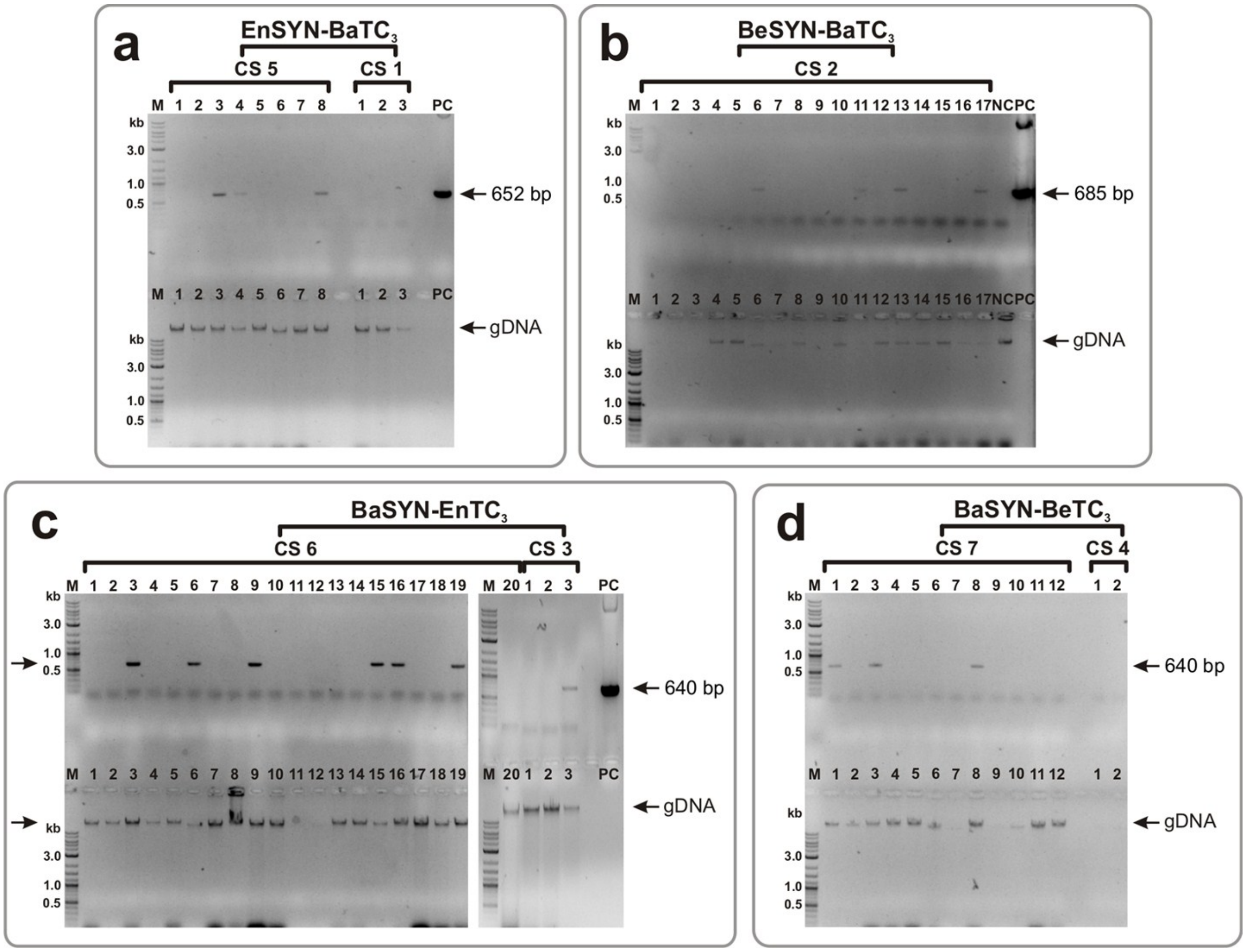

Supplementary Fig. 10: Synthetase integration into the genome of *A. niger*. Extracted gDNA of the transformants was confirmed by gel electrophoresis and scanned for synthetase integration by colony PCR. The expected band sizes are given in Supplementary Table 11. (a) EnSYN-BaTC$_3$, (b) BeSYN-BaTC$_3$, (c) BaSYN-EnTC$_3$, (d) BaSYN-BeTC$_3$. NC: Negative control, PC: Positive control.

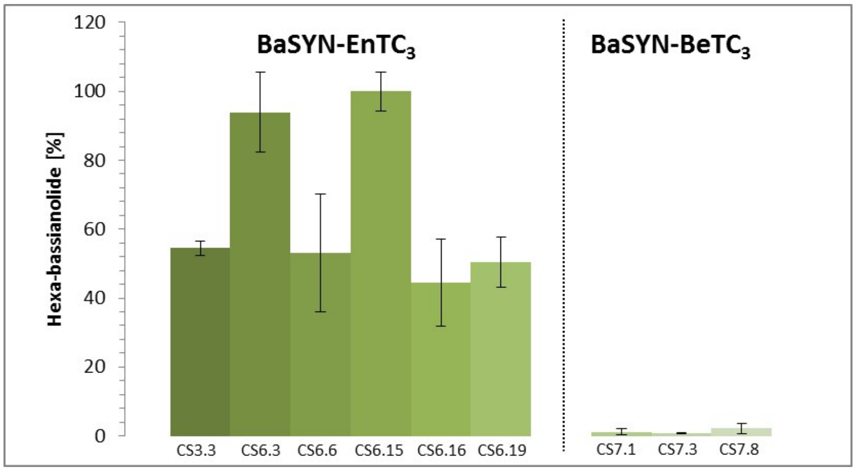

Supplementary Fig. 11: Comparison of BaSYN-EnTC$_3$ and BaSYN-BeTC$_3$ by UHPLC-ESI-MRM analysis. The relative hexa-bassianolide synthesis levels by individual *A. niger* transformants were compared on an ESI-Triple-Quadrupole mass spectrometer in MRM-mode. The highest production was set to 100 %.

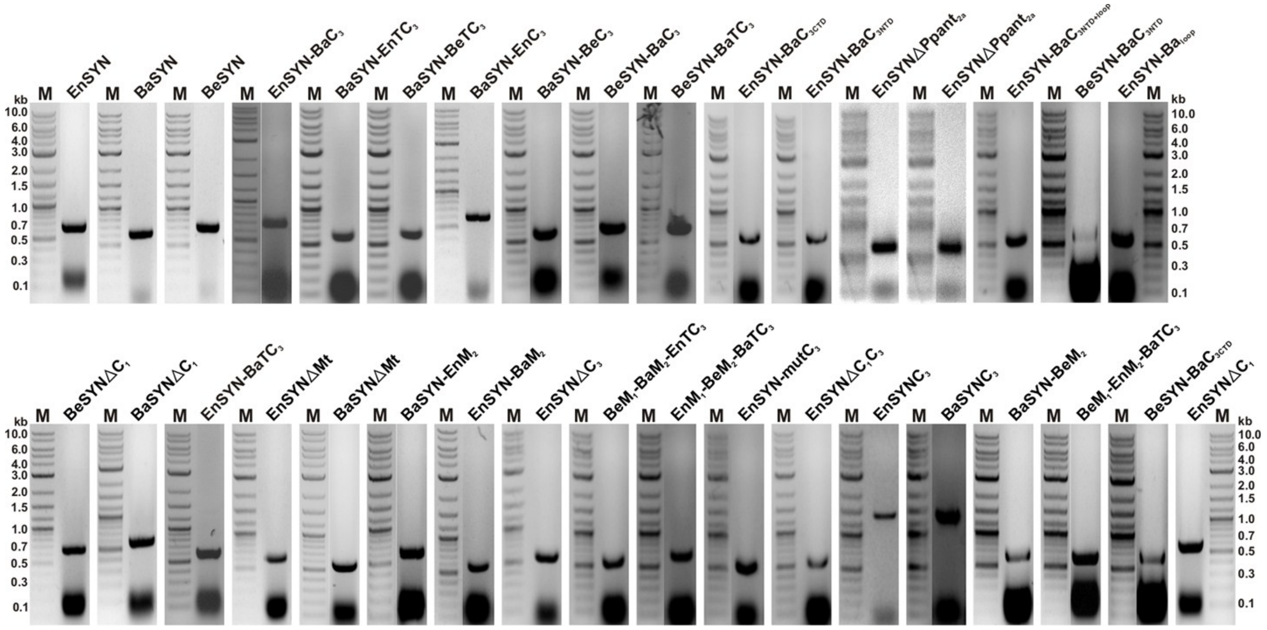

Supplementary Fig. 12: Colony PCR of chosen clones after synthetase transformation into *E. coli* DH5α (K12). The expected band sizes are given in Supplementary Table 11.

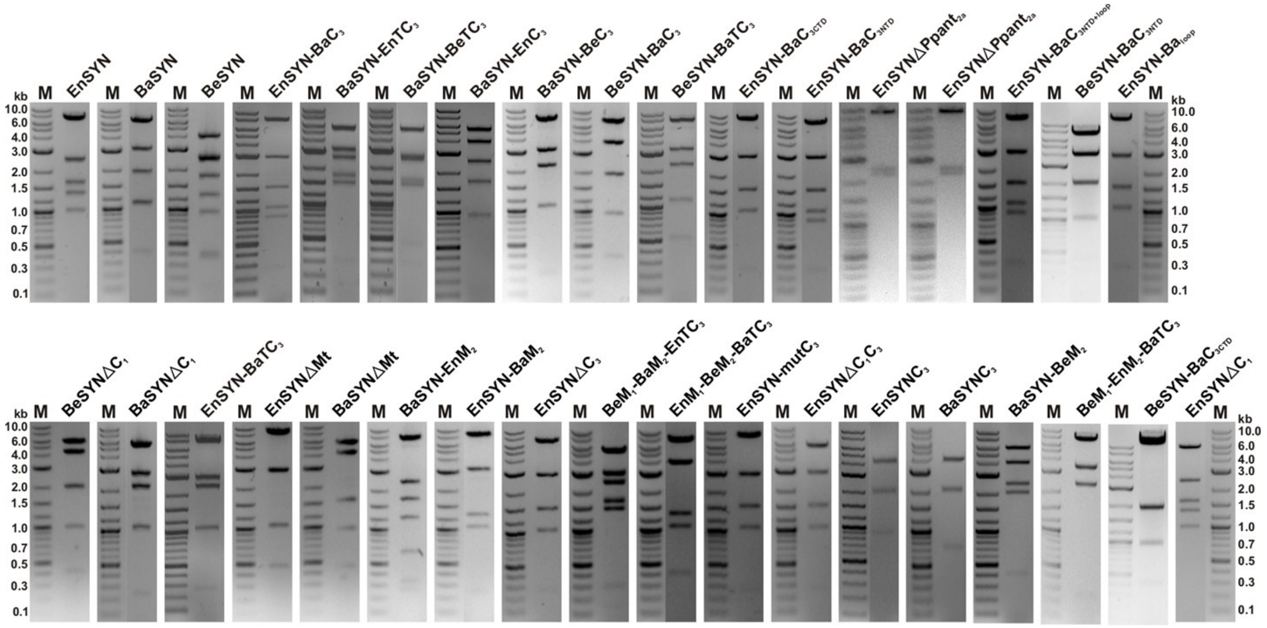

Supplementary Fig. 13: Control restriction of chosen clones after synthetase transformation into *E. coli* DH5α (K12). The expected band sizes are given in Supplementary Table 12.

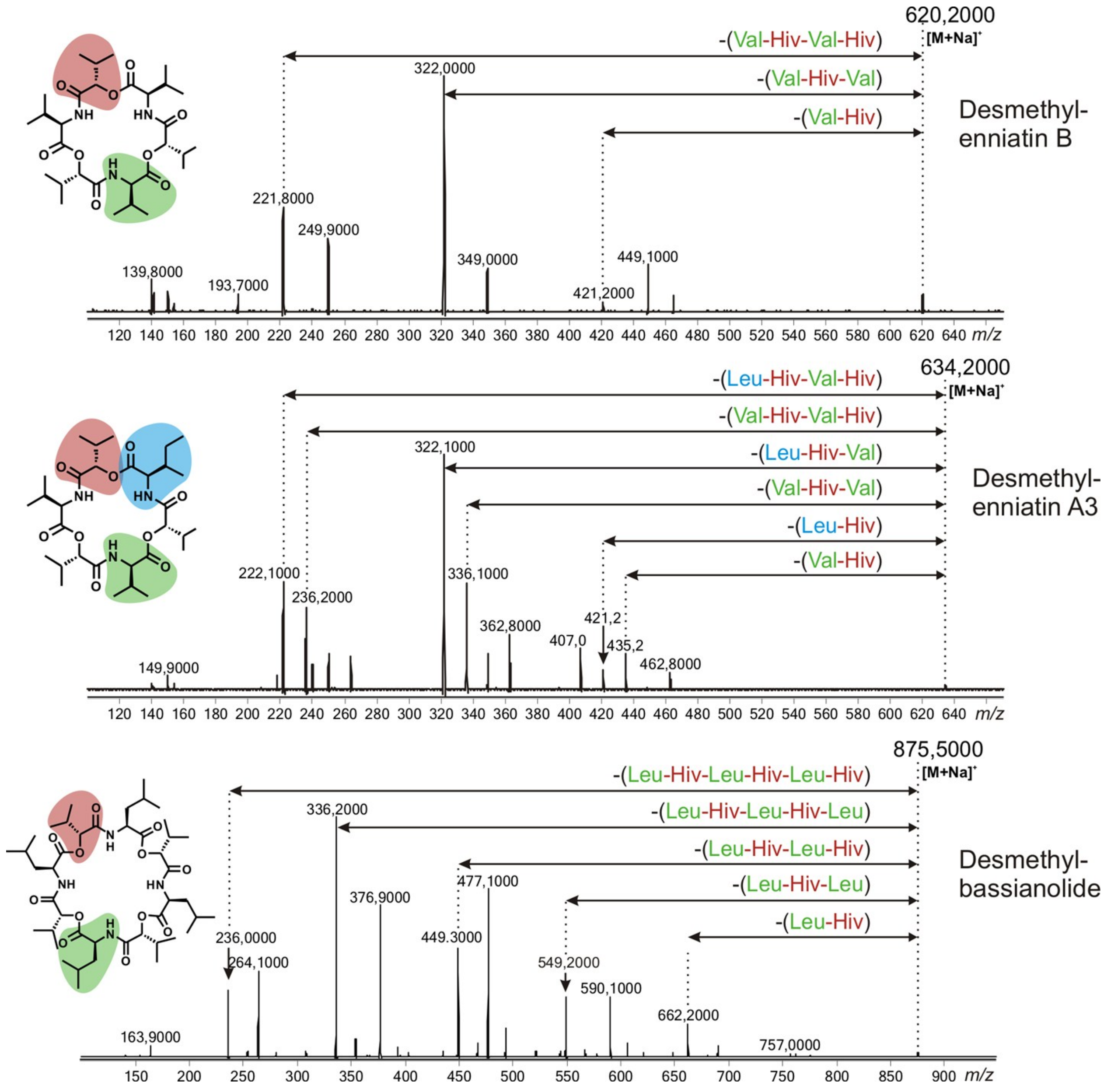

Supplementary Fig. 14: MS/MS spectra of desmethyl-cyclodepsipeptides. Analysis was performed on an ESI-Triple-Quadrupole mass spectrometer in MS/MS-mode.

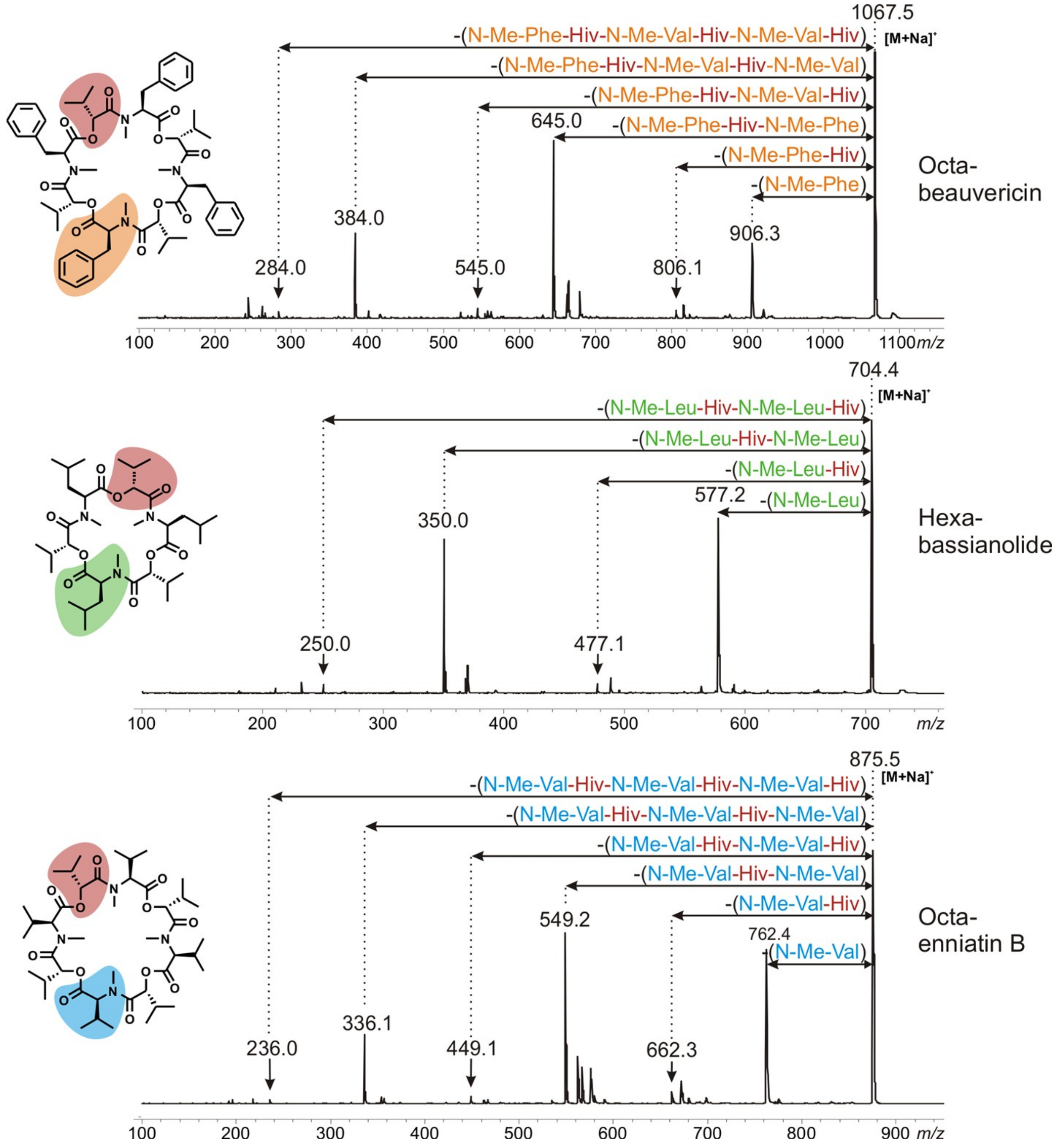

Supplementary Fig. 15: MS/MS spectra of hybrid CDPs. Analysis of octa-beauvericin, hexa-bassianolide and octa-enniatin B was performed on a MALDI-TOF(/TOF) ultrafleXtreme mass spectrometer (Bruker, Karlsruhe, Germany) in LIFT-mode.

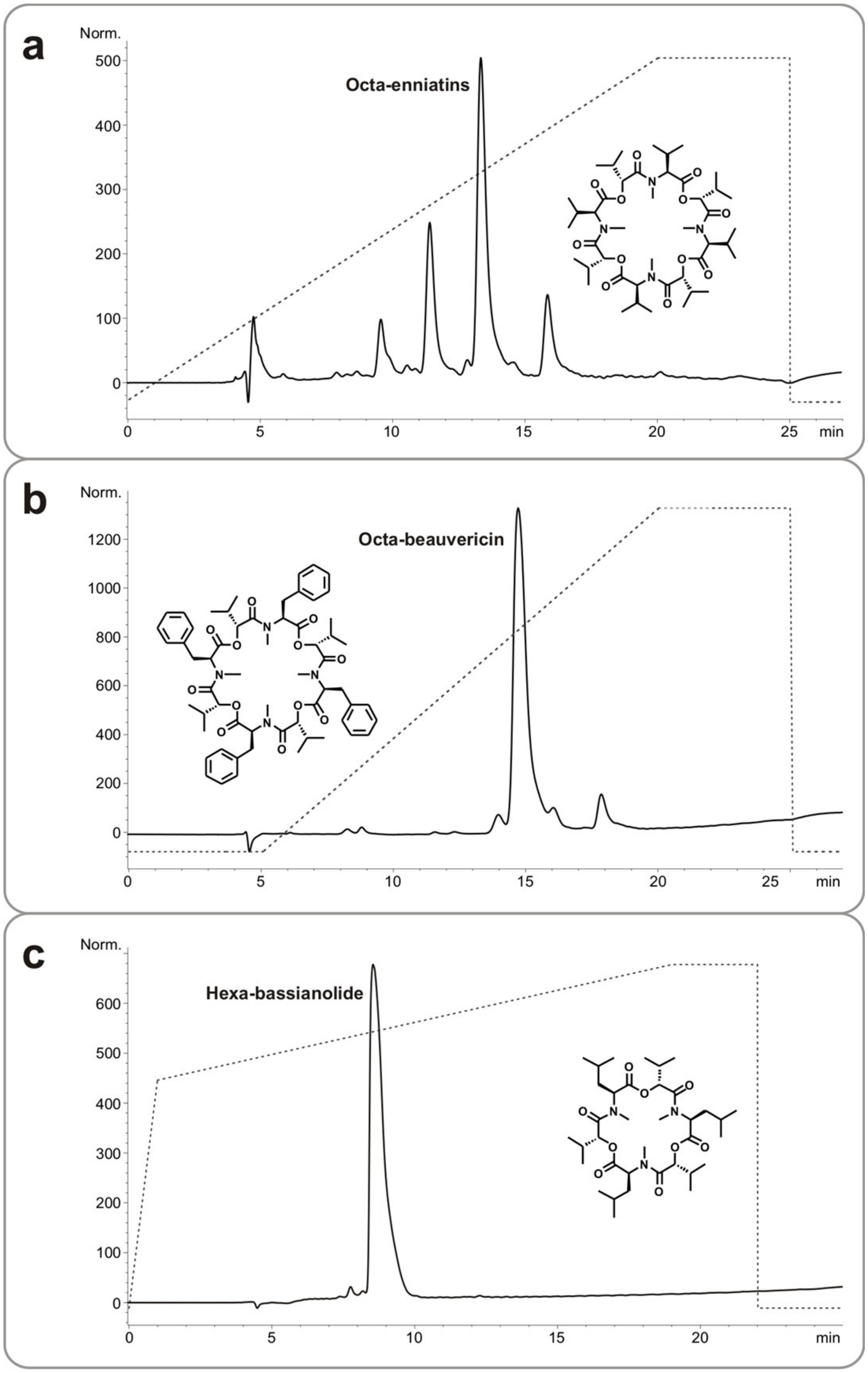

Supplementary Fig. 16: Purification of hybrid CDPs by preparative HPLC. Samples were separated on an HPLC 1100 Series (Agilent Technologies, Waldbronn, Germany; Supplementary Table 18). (a) Octa-enniatin B, (b) octa-beauvericin, (c) hexa-bassianolide.

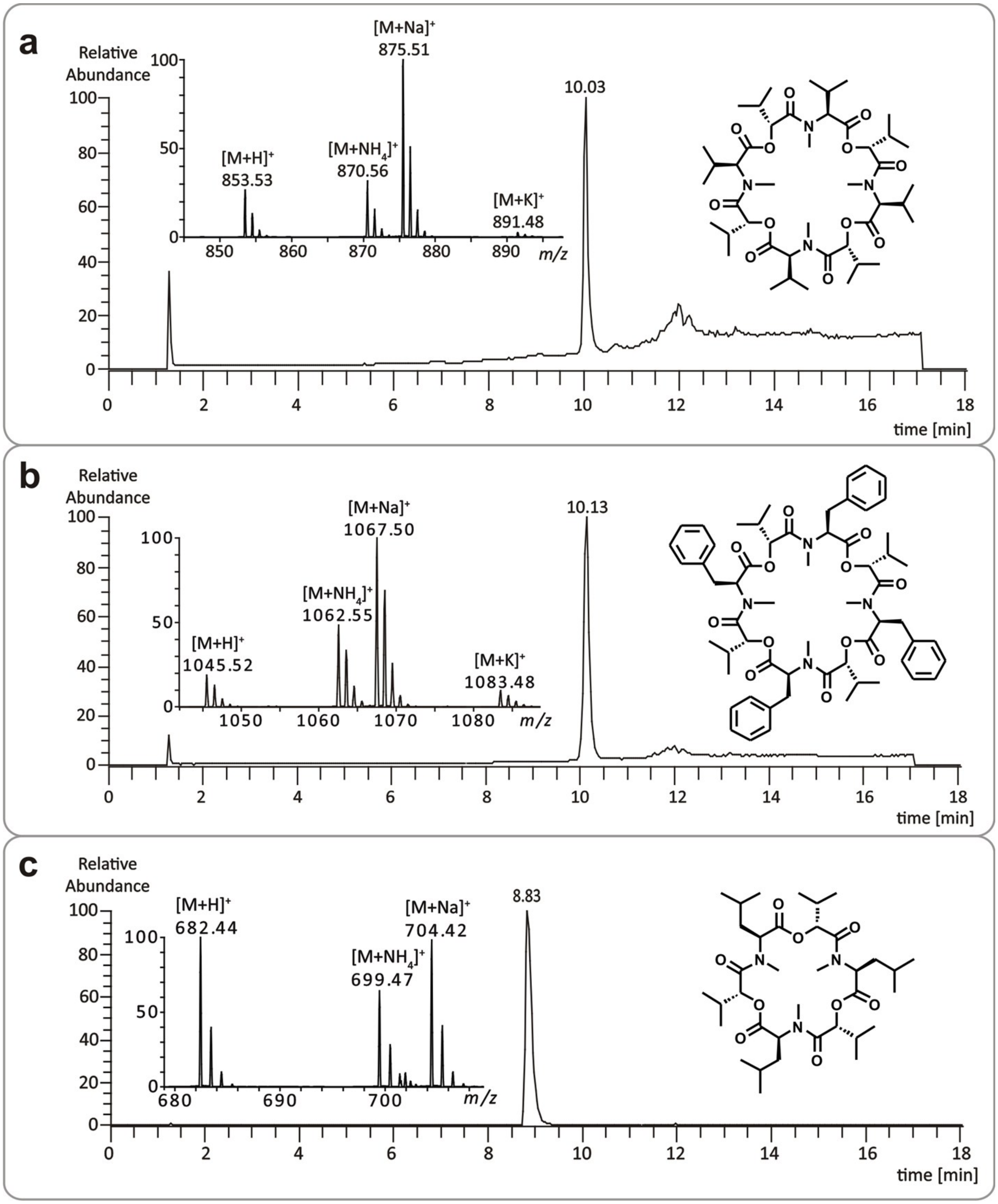

Supplementary Fig. 17: LC-ESI-MS of purified CDPs in positive ion mode. Samples were measured on an exactive mass spectrometer (Thermo Fisher Scientific, Dreieich, Germany) in MS and MS/MS-mode. (a) Octa-enniatin B, (b) octa-beauvericin, (c) hexa-bassianolide.

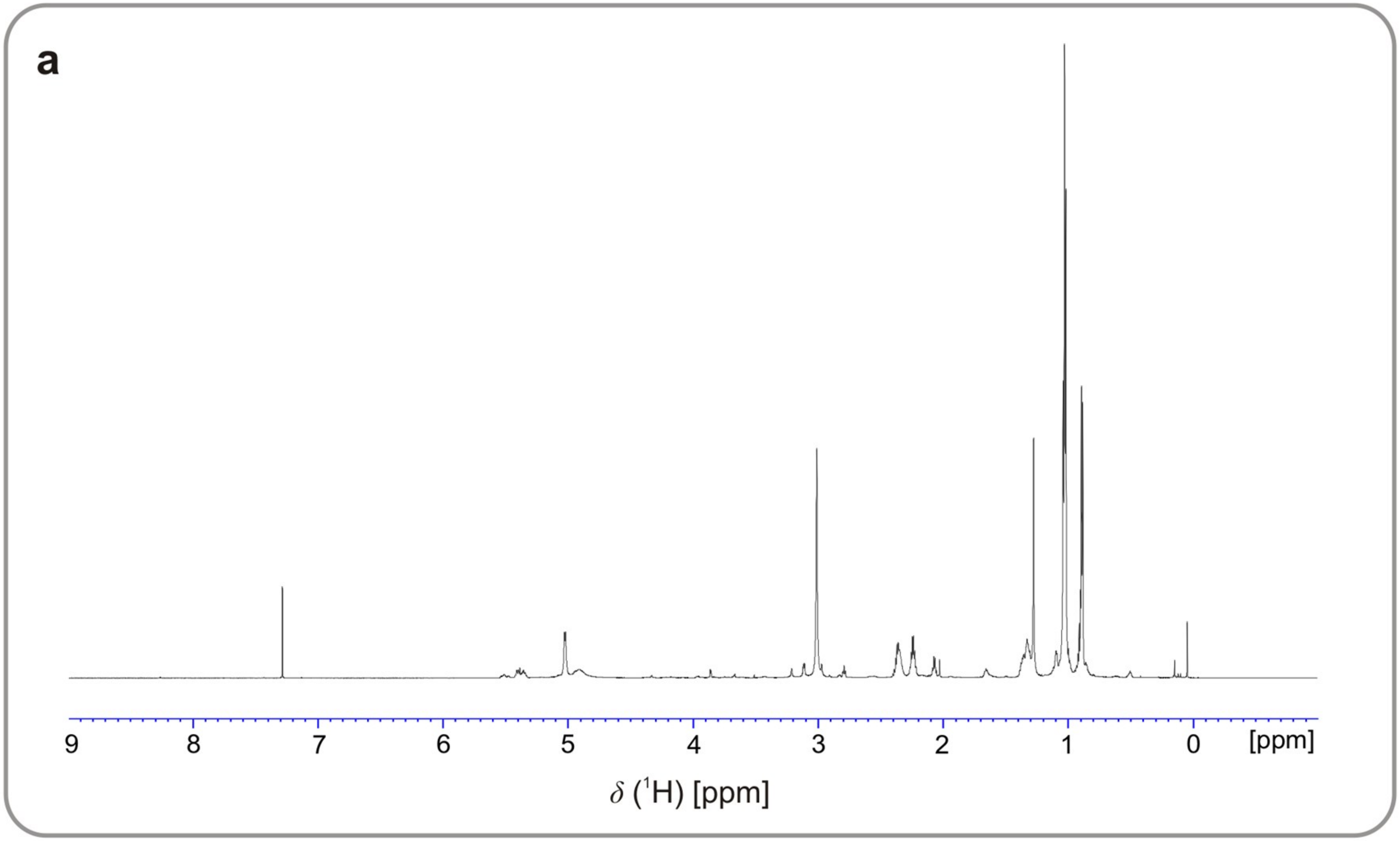
a
9
8
7
6
5
4
3
2
1
0
[ppm]
δ (1H) [ppm]

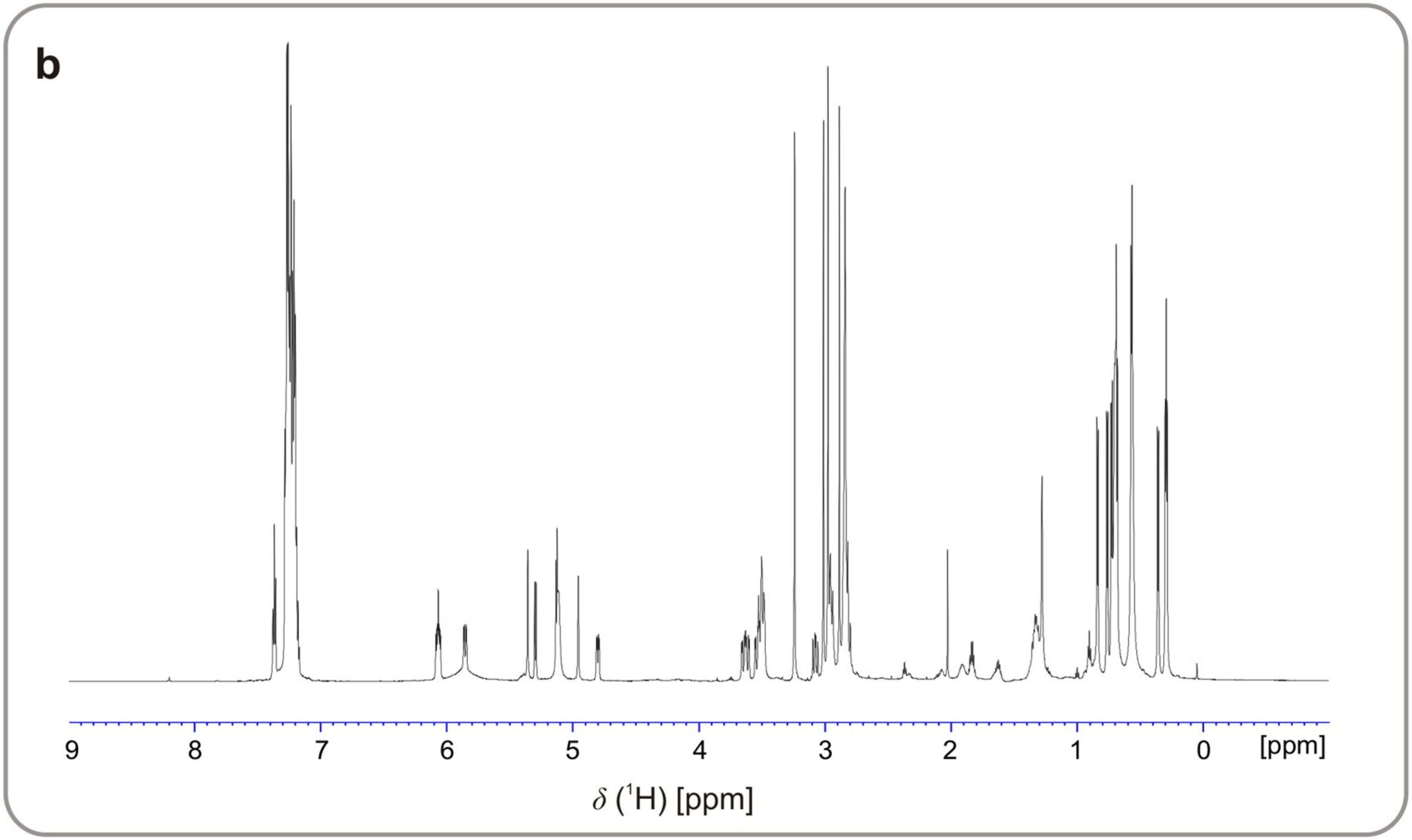
b
9
8
7
6
5
4
3
2
1
0
[ppm]
δ (¹H) [ppm]

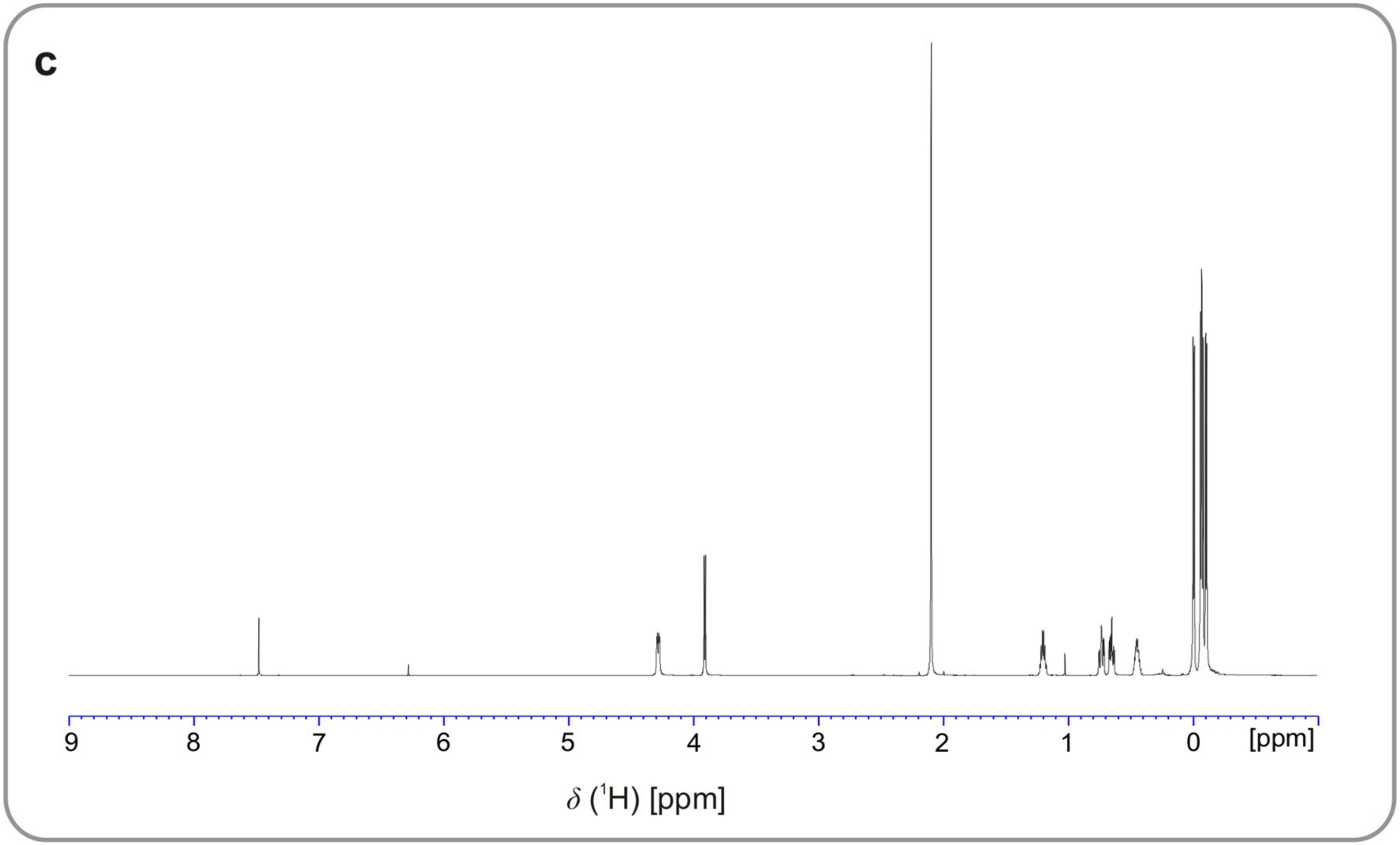

Supplementary Fig. 18: ^{1}H NMR spectra of purified hybrid CDPs. (a) Octa-enniatin B, (b) octa-beauvericin, (c) hexa-bassianolide.

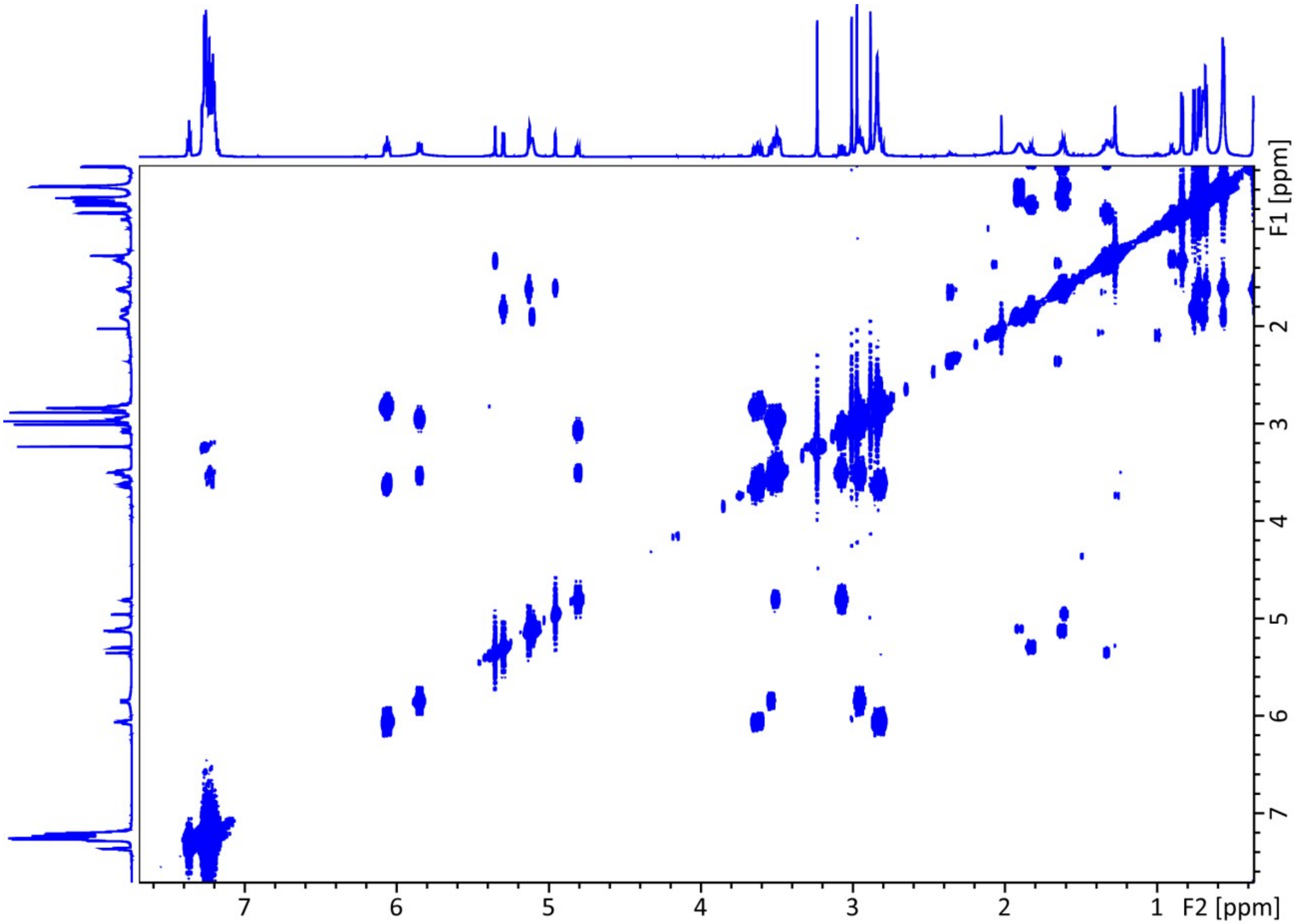

Supplementary Fig. 19: ^{1}H-^{1}H COSY spectrum of octa-beauvericin.

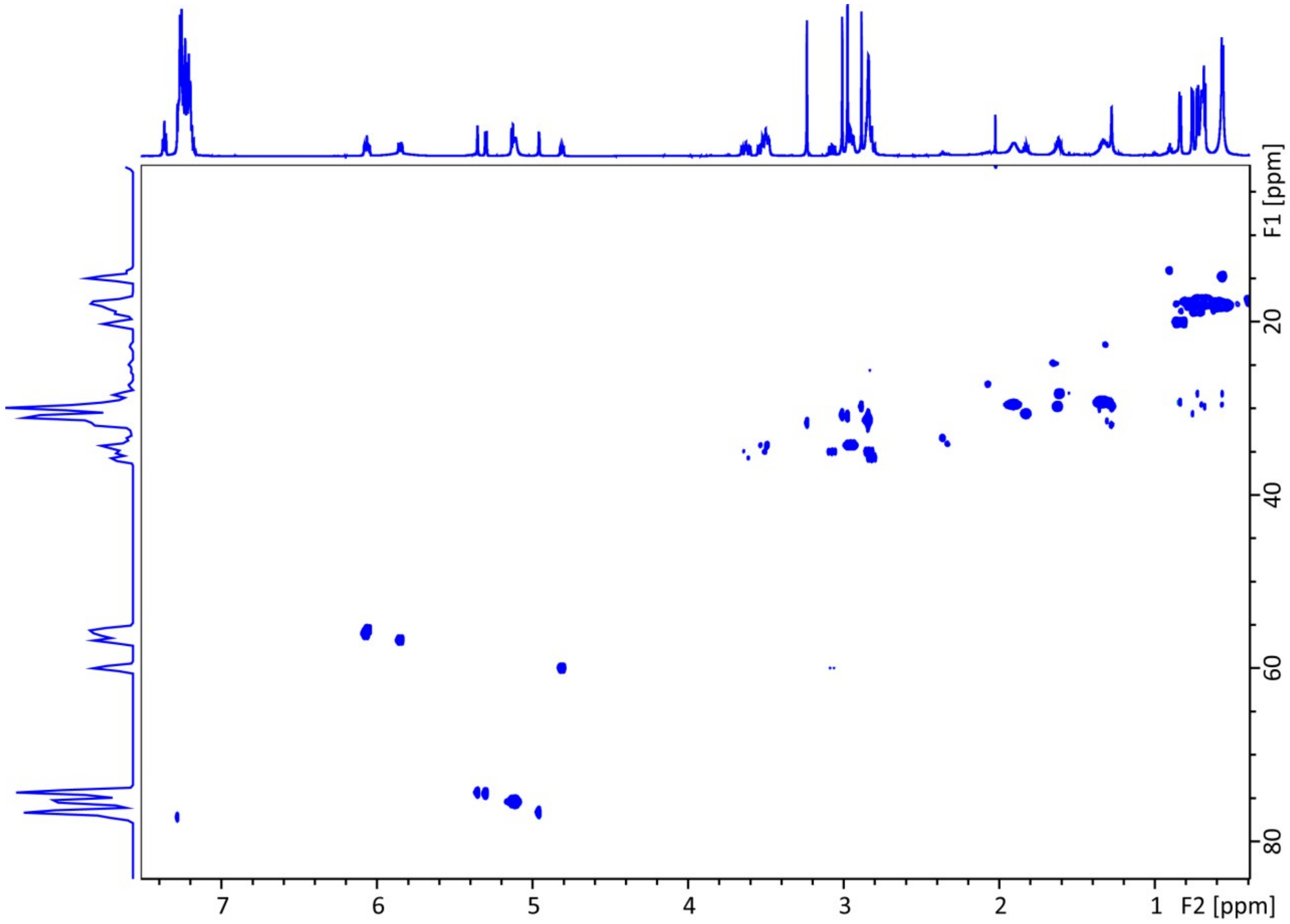

Supplementary Fig. 20: ^{1}H-^{13}C HSQC spectrum of octa-beauvericin.

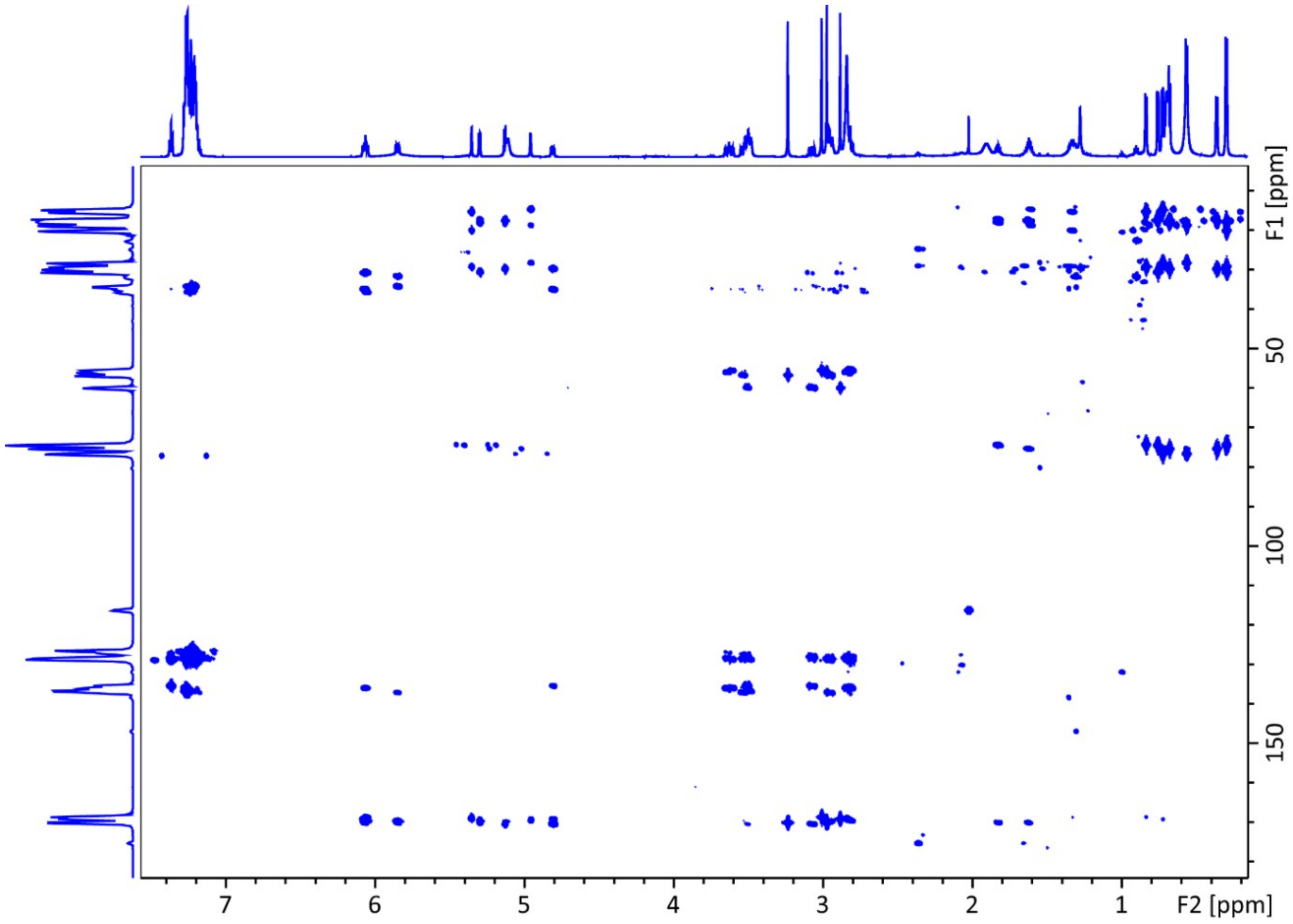

Supplementary Fig. 21: ^{1}H-^{13}C HMBC spectrum of octa-beauvericin.

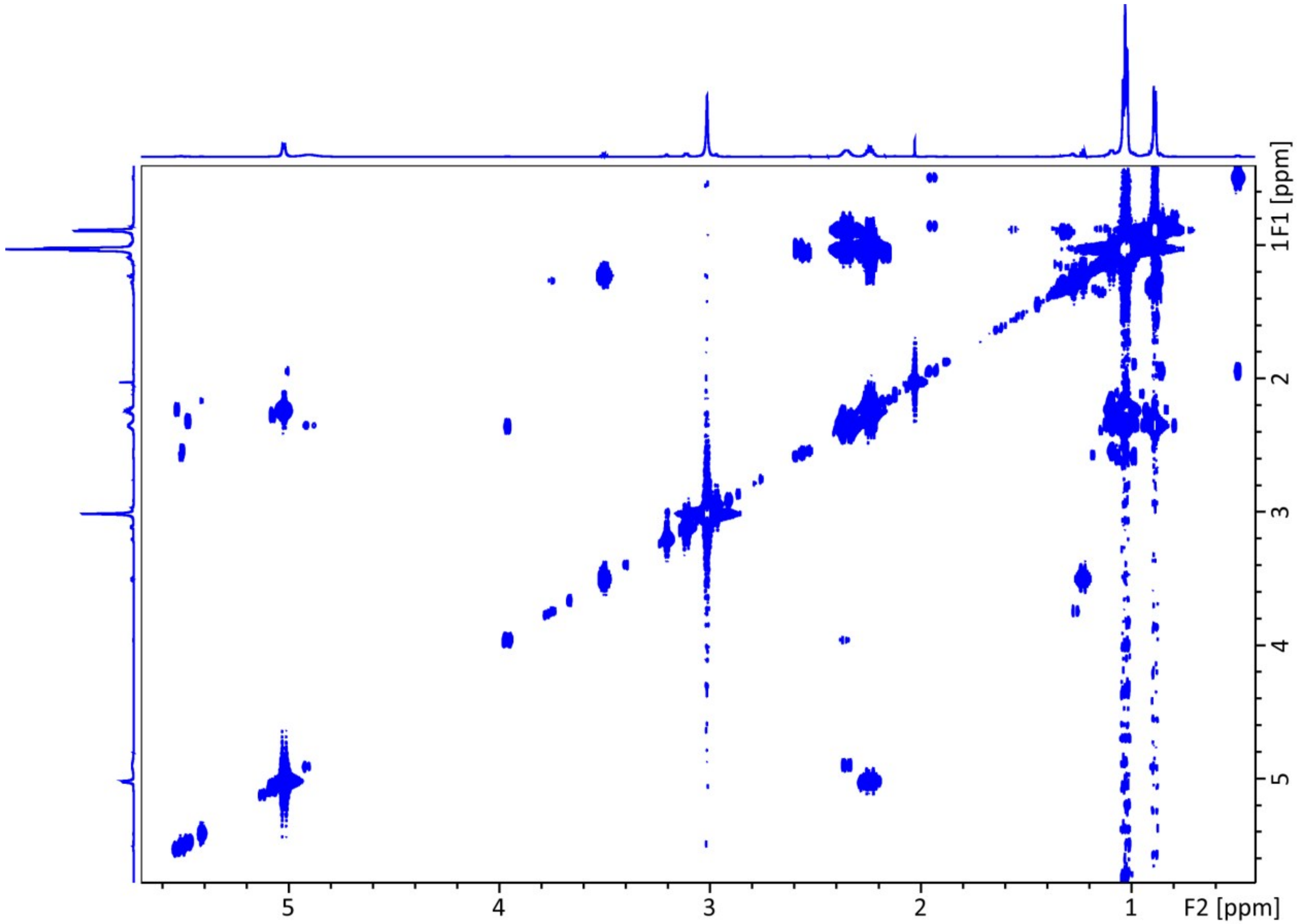

Supplementary Fig. 22: ^{1}H-^{1}H COSY spectrum of octa-enniatin B.

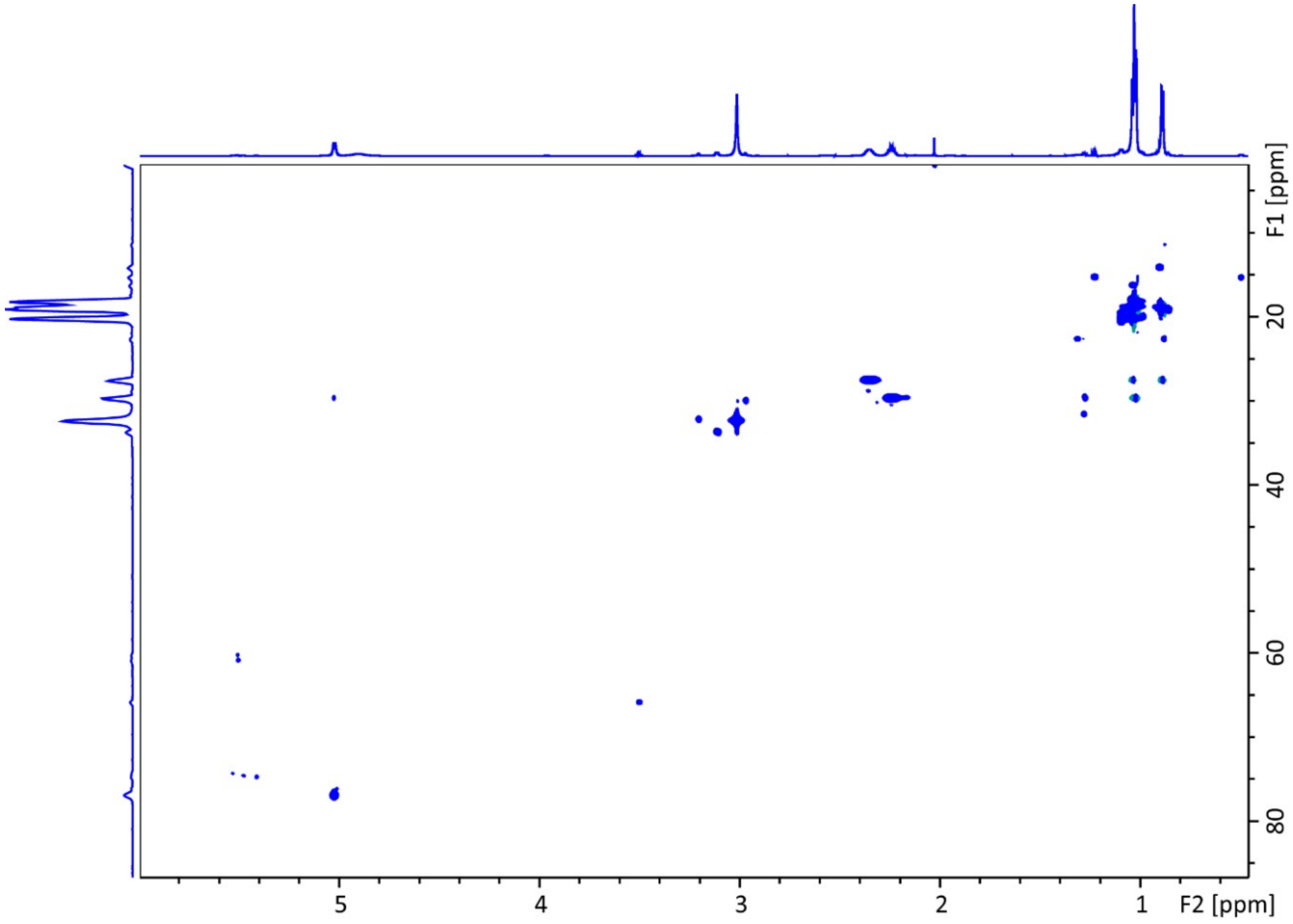

Supplementary Fig. 23: ^1H-^{13}C HSQC spectrum of octa-enniatin B.

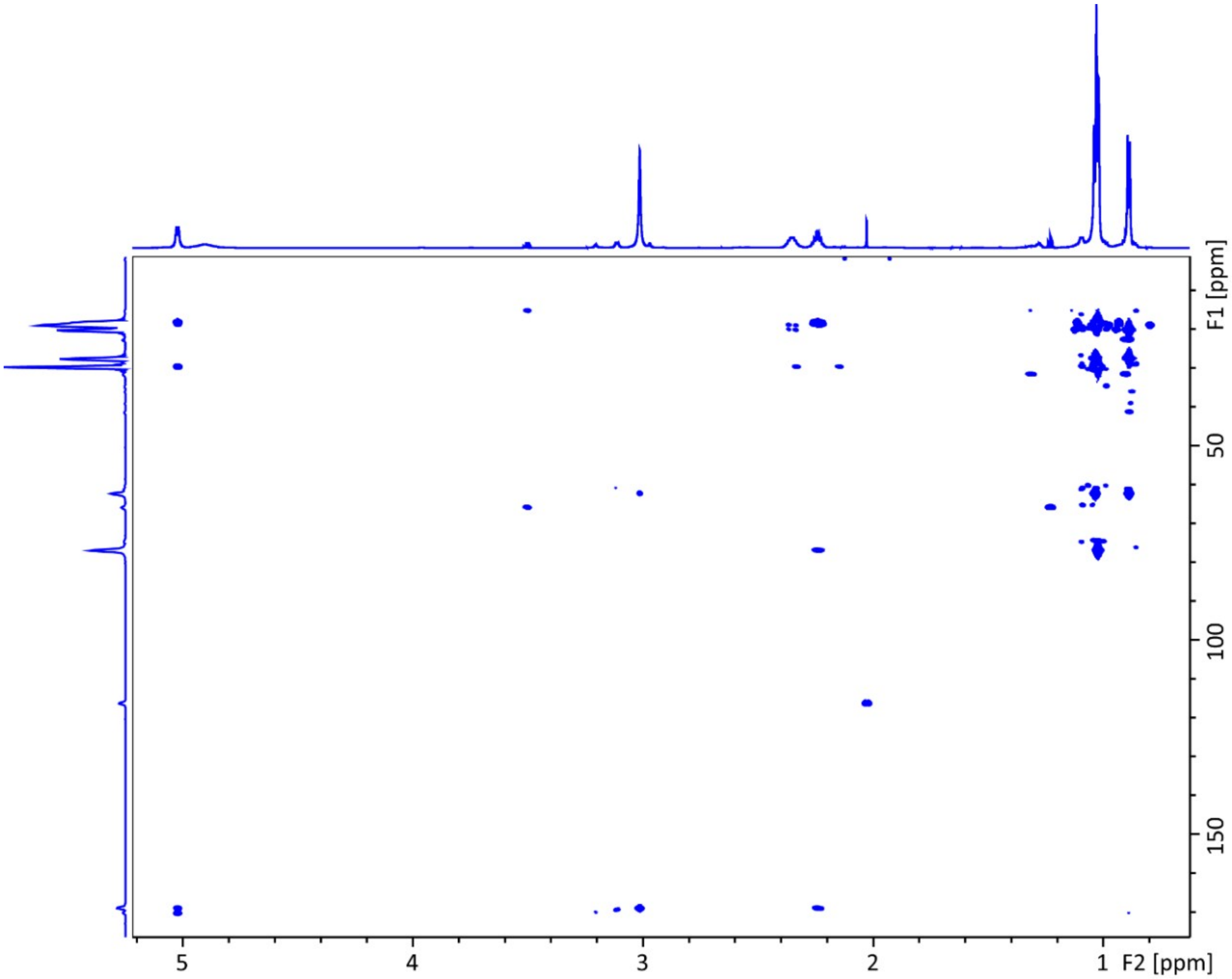

Supplementary Fig. 24: ^1H-^{13}C HMBC spectrum of octa-enniatin B.

4. References

1 L. Richter, F. Wanka, S. Boecker, D. Storm, T. Kurt, Ö. Vural, R. Süßmuth and V. Meyer, *Fungal Biol. Biotechnol.*, 2014, **1**, 4.

2 D. G. Gibson, L. Young, R. Chuang, J. C. Venter, C. A. Hutchison and H. O. Smith, *Nat. Methods*, 2009, **6**, 343–345.

3 V. Meyer, F. Wanka, J. van Gent, M. Arentshorst, C. A. M. J. J. van den Hondel and A. F. J. Ram, *Appl. Environ. Microbiol.*, 2011, **77**, 2975–2983.

4 A. Shevchenko, H. Tomas, J. Havlis, J. V Olsen and M. Mann, *Nat. Protoc.*, 2007, **1**, 2856–2860.

5 M. C. Chambers, B. Maclean, R. Burke, D. Amodei, D. L. Ruderman, S. Neumann, L. Gatto, B. Fischer, B. Pratt, J. Egertson, K. Hoff, D. Kessner, N. Tasman, N. Shulman, B. Frewen, T. A. Baker, M.-Y. Brusniak, C. Paulse, D. Creasy, L. Flashner, K. Kani, C. Moulding, S. L. Seymour, L. M. Nuwaysir, B. Lefebvre, F. Kuhlmann, J. Roark, P. Rainer, S. Detlev, T. Hemenway, A. Huhmer, J. Langridge, B. Connolly, T. Chadick, K. Holly, J. Eckels, E. W. Deutsch, R. L. Moritz, J. E. Katz, D. B. Agus, M. MacCoss, D. L. Tabb and P. Mallick, *Nat. Biotechnol.*, 2012, **30**, 918–920.

6 M. Vaudel, H. Barsnes, F. S. Berven, A. Sickmann and L. Martens, *Proteomics*, 2011, **11**, 996–999.

7 M. Vaudel, J. M. Burkhart, R. P. Zahedi, E. Oveland, F. S. Berven, A. Sickmann, L. Martens and H. Barsnes, *Nat. Biotechnol.*, 2015, **33**, 22–4.

8 S. Zobel, S. Boecker, D. Kulke, D. Heimbach, V. Meyer and R. D. Süssmuth, *ChemBioChem*, 2016, **17**, 283–287.

9 I. Orhan, B. Şener, M. Kaiser, R. Brun and D. Tasdemir, *Mar. Drugs*, 2010, **8**, 47–58.

10 R. Krieg, E. Jortzik, A.-A. Goetz, S. Blandin, S. Wittlin, M. Elhabiri, M. Rahbari, S. Nuryyeva, K. Voigt, H.-M. Dahse, A. Brakhage, S. Beckmann, T. Quack, C. G. Grevelding, A. B. Pinkerton, B. Schönecker, J. Burrows, E. Davioud-Charvet, S. Rahlfs and K. Becker, *Nat. Commun.*, 2017, **8**, 14478.
